# Whole-genome sequencing provides insights into the genetic diversity and domestication of bitter gourd (*Momordica* spp.)

**DOI:** 10.1038/s41438-020-0305-5

**Published:** 2020-06-01

**Authors:** Junjie Cui, Yan Yang, Shaobo Luo, Le Wang, Rukui Huang, Qingfang Wen, Xiaoxia Han, Nansheng Miao, Jiaowen Cheng, Ziji Liu, Changyuan Zhang, Chengcheng Feng, Haisheng Zhu, Jianwen Su, Xinjian Wan, Fang Hu, Yu Niu, Xiaoming Zheng, Yulan Yang, Dai Shan, Zhensheng Dong, Weiming He, Narinder P. S. Dhillon, Kailin Hu

**Affiliations:** 1 College of Horticulture, South China Agricultural University/State Key Laboratory for Conservation and Utilization of Subtropical Agro-bioresources/Key Laboratory of Biology and Genetic Improvement of Horticultural Crops (South China), Ministry of Agriculture and Rural Affairs, 510642 Guangzhou, China; 2 Tropical Crop Genetic Resources Institute, Chinese Academy of Tropical Agricultural Sciences, 571737 Danzhou, China; 3 Vegetable Research Institute, Guangdong Academy of Agricultural Sciences, 510640 Guangzhou, China; 4 BGI Genomics, BGI-Shenzhen, 518083 Shenzhen, China; 5 Vegetable Research Institute, Guangxi Academy of Agricultural Sciences, 530007 Nanning, China; 6 Crop Research Institute, Fujian Academy of Agricultural Sciences, 350013 Fuzhou, China; 7 Institute of Vegetable Research, Hunan Academy of Agricultural Sciences, 410125 Changsha, China; 8 Institute of Vegetables and Flowers, Jiangxi Academy of Agricultural Sciences, 330200 Nanchang, China; 9 World Vegetable Center, East and Southeast Asia, Research and Training Station, Kasetsart University, Kamphaeng Saen, Nakhon Pathom, 73140 Thailand

**Keywords:** Comparative genomics, Genome

## Abstract

Bitter gourd (*Momordica charantia*) is a popular cultivated vegetable in Asian and African countries. To reveal the characteristics of the genomic structure, evolutionary trajectory, and genetic basis underlying the domestication of bitter gourd, we performed whole-genome sequencing of the cultivar Dali-11 and the wild small-fruited line TR and resequencing of 187 bitter gourd germplasms from 16 countries. The major gene clusters (*Bi* clusters) for the biosynthesis of cucurbitane triterpenoids, which confer a bitter taste, are highly conserved in cucumber, melon, and watermelon. Comparative analysis among cucurbit genomes revealed that the *Bi* cluster involved in cucurbitane triterpenoid biosynthesis is absent in bitter gourd. Phylogenetic analysis revealed that the TR group, including 21 bitter gourd germplasms, may belong to a new species or subspecies independent from *M. charantia*. Furthermore, we found that the remaining 166 *M. charantia* germplasms are geographically differentiated, and we identified 710, 412, and 290 candidate domestication genes in the South Asia, Southeast Asia, and China populations, respectively. This study provides new insights into bitter gourd genetic diversity and domestication and will facilitate the future genomics-enabled improvement of bitter gourd.

## Introduction

Bitter gourd (*Momordica charantia*) is an economically important vegetable crop in the family Cucurbitaceae, which also includes common vegetables and fruits such as cucumber (*Cucumis sativus*), watermelon (*Citrullus lanatus*), and melon (*Cucumis melo*). Bitter gourd is native to Africa^1^ but was domesticated in Asia over a long period of time, with written Sanskrit records dating back to Indo-Aryan culture (2000 to 200 BC)^2^. *M. charantia* var. *muricata* (small fruited; hereafter, *muricata*) was first identified by Willdenow in the Hortus Malabaricus^3,4^, a book from the seventeenth century describing the flora of southern India, where other researchers later inferred it to be the wild progenitor of cultivated *M. charantia*^5–7^. However, the evolutionary trajectory and genetic basis underlying the domestication of bitter gourd remain largely unknown^8^.

Bitter gourd is a popular vegetable characterized by its bitter fruits. This bitterness is a result of cucurbitane triterpenoids, including cucurbitacins (sapogenins) and cucurbitane glycosides (saponins)^9,10^. Bitter gourd is often used in folk medicine to manage type 2 diabetes, and recent clinical studies have confirmed its role in lowering elevated fasting glucose levels in prediabetes patients^11–13^.

Although bitter gourd has been cultivated for centuries, the improvement of its varieties and cultivars has been hindered by the extreme genetic homogeneity of commercial varieties, as well as the low-genetic diversity in natural populations^14^; therefore, there is great demand for genetic resources that can improve bitter gourd varieties.

Recently, a draft genome sequence of the bitter gourd line *Momordica charantia* OHB3-1 was reported, with a scaffold-level genome assembly of 285.5 Mb and 45,859 protein-coding genes annotated by ab initio prediction^15^. However, a more accurately annotated chromosome-level genome assembly for bitter gourd is still necessary. Population-scale genomic variation analysis by resequencing has been shown to be a powerful approach for revealing the genetic diversity and genetic basis underlying domestication in many crops, including rice^16^, maize^17^, soybean^18^, cucumber^19^, and tomato^20^. However, to our knowledge, no studies have investigated population-scale genomic variation in bitter gourd.

Here, we report high-quality genome sequences for bitter gourd. In addition, we resequenced 187 bitter gourd germplasms from a worldwide collection, as well as one *M. balsamina* and one *M. foetida* accession. Our data provide an improved understanding of bitter gourd diversity and domestication, paving the way for efficiently breeding new bitter gourd cultivars.

## Materials and methods

### Sample collection and genome sequencing

De novo whole-genome sequencing was conducted in two bitter gourd lines, *M. charantia* Dali-11 collected from Foshan city, Guangdong Province, China, and the small-fruited line TR collected from Singida, Tanzania. For Dali-11, libraries with an increasing gradient of insert sizes of 170 bp, 500 bp, 800 bp, 2 kb, 5 kb, 10 kb, and 20 kb were constructed and sequenced on the Illumina HiSeq 2000 platform. Nine paired-end libraries were generated, and 12 lanes were sequenced, producing 92.46 Gb of raw data. Low-quality reads, including short-insert library reads comprising 40% of bases with quality scores ≤7 and large-insert library reads that comprising >35% of bases with quality scores ≤7, were filtered out, as were PCR duplicates in which read1 and read2 of two paired-end reads were completely identical. The filtration of low-quality and duplicated reads resulted in 75.31 Gb (~251× coverage) of data for genome assembly. For TR, six paired-end libraries with insert sizes of 270 bp, 800 bp, 2 kb, 5 kb, and 10 kb were prepared. In total, ~70.55 Gb and 55.68 Gb (~185× coverage) of raw data and clean data were generated, respectively, for subsequent genome assembly.

The samples that were resequenced were as follows: 166 *M. charantia* (including 136 intermediate-size- to large-fruited *M*. *charantia* and 30 small-fruited *muricata*) samples, 21 small-fruited TR-group samples, one *M. balsamina* line, and one *M. foetida* line (Supplementary Table S27). Sequencing libraries were constructed according to the manufacturer’s instructions (Illumina). Short reads were generated by applying the SolexaPipeline-0.3 base-calling pipeline (Illumina). Approximately 10–38x coverage of the genome sequences from each sample was generated.

### Genome assembly

After correction and filtering for short-read sequences, the bitter gourd genomes were assembled using SOAPdenovo^21^. Contigs were constructed using paired-end reads of short-insert-size libraries, and the contigs were connected using long-insert-size libraries to generate scaffolds. All reads were used to fill gaps in the scaffolds. To assemble the Dali-11 genome, scaffolds were anchored to pseudochromosomes through a high-quality RAD genetic map^22^.

The quality and completeness of our assemblies were assessed according to the following methods. First, all clean reads were mapped to the corresponding assembly to investigate the completeness of the assemblies, which can be reflected by the mapping ratio obtained using SOAP2^23^ with default parameters, and SOAPcoverage 2.27 (http://soap.genomics.org.cn/) was then used to calculate sequencing depth. Second, we searched for conserved genes by using BUSCO^24^ (http://busco.ezlab.org/) software. In addition, we de novo assembled the transcriptome data of six tissues, including roots, stems, leaves, male flowers, ovaries, and fruits, into unigenes and then mapped them back to the bitter gourd genomes.

### Transcriptome analysis

To generate transcriptomes, total RNA was extracted from Dali-11 roots, stems, leaves, male flowers, ovaries, and fruits from four developmental stages (6, 12, 18, and 24 days after pollination) using TRIzol reagent (Invitrogen, Carlsbad, CA) following the manufacturer’s instructions. The raw transcriptome reads containing adaptors or >10% unknown nucleotides and those showing low quality (>50% bases with a quality value ≤5) were filtered, and the clean reads were then mapped to the Dali-11 reference gene using Bowtie2^25^ and to the genome using TopHat^26^. The expression level for individual genes was quantified according to fragments per kilobase of exon per million reads mapped (FPKM) values using RSEM^27^.

### Genome annotation

Repetitive sequences in the bitter gourd genomes were identified using a combination of TRF^28^, Repbase-based^29,30^ and de novo methods. Three de novo analysis programs, including LTR-FINDER^31^, PILER^32^, and RepeatScout^33^, were used to generate the initial repeat library. Then, the de novo library was analyzed using RepeatMasker to annotate and classify repeats.

For gene annotation, we used homology, ab initio prediction, and transcript data to predict gene structure in the Dali-11 genome. The homology approach involved mapping protein sequences from three other cucurbit species (*C. lanatus*, *C. sativus*, and *C. melo*) to the Dali-11 genome using TBLASTN^34^ (*E*-value < 1e-5), and the homologous genome sequences were aligned against the matching proteins using GeneWise^35^. TopHat and Cufflinks were used to obtain transcript structures from RNA-seq data from the various tissues and developmental stages. Augustus (augustus-3.0.3) was employed for ab initio gene prediction^36^. GLEAN^37^ was used to merge the results from the homology and transcript analysis to form a comprehensive and nonredundant reference gene set. The genes in the TR genome were predicted using the homology-based and de novo methods described above. Once gene structures were identified in Dali-11 and TR, gene functions were assigned based on the best alignment attained using BLASTP against the Nr, SWISS-PROT^38^, TrEMBL^38^, GO^39^, KEGG^40^, and InterProScan^41^ databases.

### Genome evolution analysis

The distribution of orthologous gene families in *M. charantia* (Dali-11), *C. lanatus*, *C. melo*, *C. sativus*, *C. pepo*, *C. maxima*, *C. moschata*, *L. siceraria*, and *J. regia* was defined using OrthoMCL^42^. The resulting 2248 shared single-copy genes were used to generate the phylogeny of *M. charantia* (Dali-11) and the eight other plant species. Divergence time estimations between species were determined using MCMCtree in PAML (v4.5)^43^. The divergence time of ~84 Mya between Fagales and Cucurbitales indicated by fossil information^44^, as well as two calibrated divergence times, 26.28 Mya between *C. moschata* and *C. lanatus*^45^ and 10.10 Mya between *C. melo* and *C. sativus*^46^, were used to estimate the divergence time in this study.

Paralogous genes were detected using the all-versus-all BLASTp method (*E*-value < 1e-5), and homologous blocks were detected using MCScanX^47^. Fourfold degenerate sites (4DTv) values were calculated on the basis of concatenated nucleotide alignments with HKY substitution models^48^.

### SNP and InDel detection

Paired-end reads (clean reads) were mapped to the Dali-11 and TR genomes using BWA^49^, which resulted in a BAM file. SAMtools Picard and GATK^50,51^ were used for further handling procedures such as alignment, repeat removal, and ID addition. The GATK pipeline was used to detect SNPs and InDels for each sample. Small insertions and deletions (≤50 bp in length) were identified in this study.

### Population analysis

Three SNP matrixes (including two separated and one combined SNP set called from the genomes of Dali-11 and TR) were used to construct neighbor-joining phylogenetic trees with PHYLIP 3.69 (http://evolution.genetics.washington.edu/phylip.html). Bootstrap values were calculated with VCF2Dis software (https://github.com/BGI-shenzhen/VCF2Dis). Principal component analysis (PCA) was performed using the EIGENSTRAT stratification correction method^52^, and the population structure was estimated using FRAPPE^53^ with calculated *K* values ranging from two to five.

The correlation coefficient (*r*^2^) of alleles was calculated to measure the level of linkage disequilibrium (LD) using PopLDdecay (https://github.com/BGI-shenzhen/PopLDdecay). The LD blocks were analyzed with Haploview^54^.

The genetic separation between individual genomes was inferred via the multiple sequentially Markovian coalescent (MSMC) method^55^, with a generation time of 1 year and a rate of 1.0 × 10^-8^ mutations per nucleotide per generation^56^. We also measured nucleotide diversity (*θπ*), Watterson’s estimator (*θw*)^57^, Tajima’s *D*^58^, and Wright’s fixation index (*F*_ST_)^59^ in or between different bitter gourd populations according to the corresponding formulas.

To identify the regions underlying the genetic changes caused by different geographic areas of domestication, 30 wild (*muricata*) samples (Wild30) and groups of 30 large-fruited bitter gourd samples from South Asia (SA30), Southeast Asia (SEA30), and China (CHIN30) were selected. The diversity ratios and cross-population composite likelihood ratios (XP-CLR)^60^ between SA30, SEA30, and CHN30 and Wild30 were calculated, and regions were identified as domestication regions when both the *π* (*θπ*) values and XP-CLR ratios were in the top 5% of the distribution outliers.

## Results

### Sequencing and de novo assembly of the bitter gourd genomes

We performed whole-genome sequencing of the bitter gourd cultivar Dali-11 (*M. charantia*) from Guangdong, China, and the wild small-fruited line TR from Singida, Tanzania (Supplementary Figs. S1 and S2). Both lines had an estimated genome size of approximately 300 Mb, which was lower than the 339 Mb genome size of OHB3-1 and that of other cucurbits (Table 1 and Supplementary Figs. S3 and S4, Supplementary Table S1). After filtering, we generated a total of 75.3 Gb (251.0×) and 55.7 Gb (185.0×) of high-quality genomic reads for Dali-11 and TR, respectively (Supplementary Tables S2 and S3). The resulting de novo assembly contained 293.6 and 296.3 Mb scaffolds for Dali-11 and TR, with N50 lengths of 3.3 and 0.6 Mb, respectively (Table 1 and Supplementary Tables S4 and S5). We mapped all clean reads back to the Dali-11 assembly. The mapping ratios of all short- and large-insert-size libraries were 94.80% and 82.65%, respectively (Supplementary Table S6), and the assembly contained 96.2% of the 59,740 unigenes assembled from the transcriptome sequences of various tissues (Supplementary Tables S7 and S8). As a new draft genome, the Dali-11 assembly exhibited more complete BUSCOs (96.7%) than the OHB3-1 assembly (95.8%) (Supplementary Table S9). Using the newly developed RAD genetic map^22^, a total of 113 scaffolds (~90% of the scaffolds were larger than 1 Mb), covering ~85.5% (251.3 Mb) of the Dali-11 assembly (Supplementary Figs. S5 and S6), were anchored to 11 pseudochromosomes (MC01 to MC11). Among the 113 scaffolds, 80 were oriented by at least two markers (Supplementary Table S10).

**Table 1:** Statistics for the bitter gourd genome assembly and annotation

Assembly	Dali-11 (*M. charantia*)	TR (*Momordica* sp.)	OHB3-1 (*M. charantia*)	Cucumber (*C. sativus*)	Watermelon (*C. lanatus*)	Melon (*C. melo*)
Estimated genome size (Mb)	300	301	339	367	425	450
Sequence depth (×)	251.0	185.0	110.0	72.2	108.6	13.5
Assembled genome size (Mb)	293.6	296.3	285.5	243.5	353.5	375.0
Anchored scaffolds (Mb)	251.3	–	172.0	177.3	330.0	316.3
Sequences anchored on chromosomes (%)	85.5%	–	60.2%	72.8%	93.5%	87.5%
N50 of scaffolds (Mb)	3.3	0.6	1.1	1.1	2.4	4.7
N50 of contigs (Kb)	62.6	16.1	–	19.8	26.4	18.2
GC content (%)	35.4	35.1	36.4	32.2	32.8	33.2
Repeat rate (%)	41.5	39.9	34.7	20.8	39.8	35.4
LTR rate (%)	31.8	33.1	27.4	11.5	30.5	25.0
Number of gene models	26,427	28,827	45,859	26,682	23,440	27,427

*Note*: The *M. charantia* OHB3-1 scaffolds were anchored to pseudochromosomes according to a previous report^15^. Excluding 26 chimeric scaffolds, 229 out of 255 scaffolds were anchored

### Repeat sequence and protein-coding gene annotation

We found that ~41.5% (121.8 Mb) and 39.9% (118.2 Mb) of the Dali-11 and TR assemblies consisted of transposable elements (TEs), among which, 31.8% and 33.1% were long-terminal repeat (LTR) retrotransposons (Table 1 and Supplementary Tables S11–S14). The bitter gourd genome has apparently accumulated more LTR retrotransposons over the past 4 million years compared to the cucumber, watermelon, and melon genomes (Supplementary Fig. S7). To facilitate gene annotation, we generated ~537 million clean transcriptome reads from the roots, stems, leaves, flowers, and fruit tissue of Dali-11 (Supplementary Table S7). Using an integrated method (transcriptome, homology-based, and ab initio approaches), we predicted 26,427 high-confidence protein-coding genes in the Dali-11 genome (Supplementary Tables S15 and S16). For the TR genome, we annotated 28,827 protein-coding genes by using the homology-based and ab initio approaches (Supplementary Tables S17 and S18). The number of genes predicted in both bitter gourd genomes was close to that in the cucumber, watermelon, and melon genomes but much lower than that in the OHB3-1 genome (Table 1). The comparative analysis of gene completeness showed that the complete BUSCO ratio of *M. charantia* Dali-11 (95.9%) and TR (95.5%) was higher than those of *M. charantia* OHB3-1 (82.20%), *C. lanatus* (86.50%), *C. melo* (86.9%), *Cucurbita pepo* (92.8%), *C. sativus* (94.8%), and *Lagenaria siceraria* (88.2%) but comparable to those of *Cucurbita maxima* (95.70%) and *Cucurbita moschata* (95.8%) (Supplementary Table S9). Approximately 85.2% and 85.5% of the predicted Dali-11 and TR genes, respectively, were functionally annotated (Supplementary Tables S19 and S20).

### Genome comparison within the Cucurbitaceae family

**Fig. 1:**
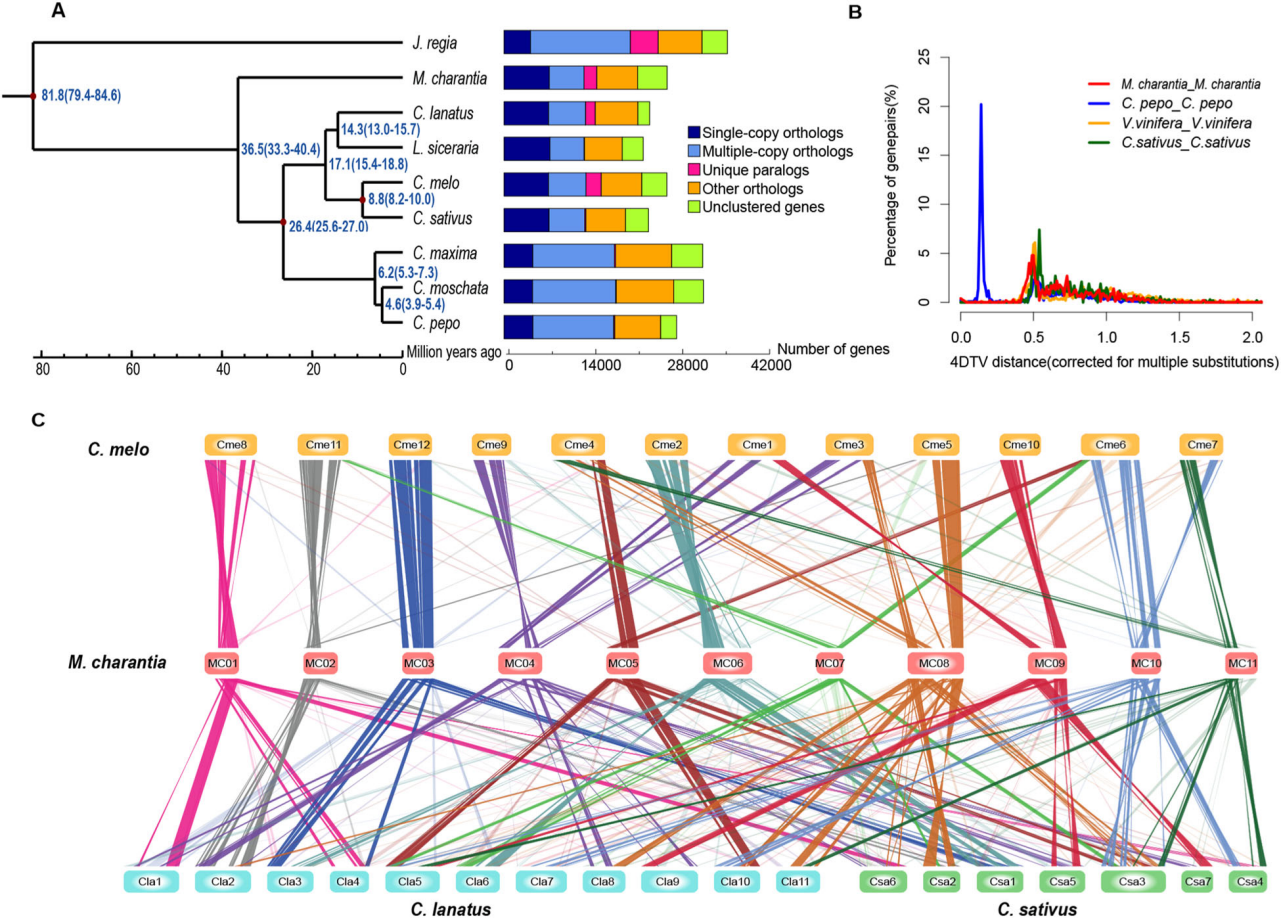
Comparative analysis and evolution of the *M. charantia* genome. **a** Phylogeny and divergence times of nine plant species (*M. charantia*, *C. lanatus*, *C. melo*, *C. sativus*, *C. pepo*, *C. maxima*, *C. moschata*, *L. siceraria*, and *J. regia*) and clusters of orthologous and paralogous gene families in the nine plant species identified by OrthoMCL. **b** The 4DTv distribution of duplicate gene pairs in *M. charantia*, *C. pepo*, *C. sativus*, and *V. vinifera*. **c** Syntenic blocks between the *M*. *charantia* genome and the three other cucurbit genomes, where syntenic blocks are defined as regions with >20 genes

In total, 2248 single-copy orthologous genes were identified in cucumber (*C. sativus*)^61^, melon (*C. melo*)^62^, watermelon (*C. lanatus*)^63^, bitter gourd (*M. charantia*), zucchini (*C. pepo*)^64^, pumpkin (*C. maxima* and *C. moschata*)^45^, bottle gourd (*L. siceraria*)^65^, and walnut (*Juglans regia*)^66^ (Fig. 1a). Phylogeny and molecular clock analysis based on the 2248 shared single-copy genes indicated that according to our species sampling, *M. charantia* split from the distantly related genus *Cucurbita* approximately 36.5 million years ago (Mya) (Fig. 1a), indicating that it is an older species compared to other cucurbit crops^67^. Similar to cucumber^61^, melon^62^, and watermelon^63^, no recent whole-genome duplication (WGD) has occurred in the *M. charantia* genome based on the distribution of 4DTv (Fig. 1b). Via genome synteny analysis, we identified 992, 807, and 922 large syntenic blocks, and these syntenic regions contained 14,938, 14,567, and 14,804 genes in *C. lanatus*, *C. melo*, and *C. sativus*, respectively (Fig. 1c and Supplementary Table S21). Moreover, we identified 22,507 gene families (ORTHOMCL clusters) in bitter gourd and eight other plant species (Supplementary Tables S22–S24), and 468 gene families containing 2,071 genes were unique to the bitter gourd genome (Supplementary Tables S25 and S26). With the exception of the annotated genes that were particularly overrepresented in the pathways of oxidative phosphorylation (ko00190), starch and sucrose metabolism (ko00500), and plant-pathogen interaction (ko04626), most of the unique bitter gourd genes had unknown functions.

### Phylogenetic analysis identifies a new species or subspecies of bitter gourd

**Fig. 2:**
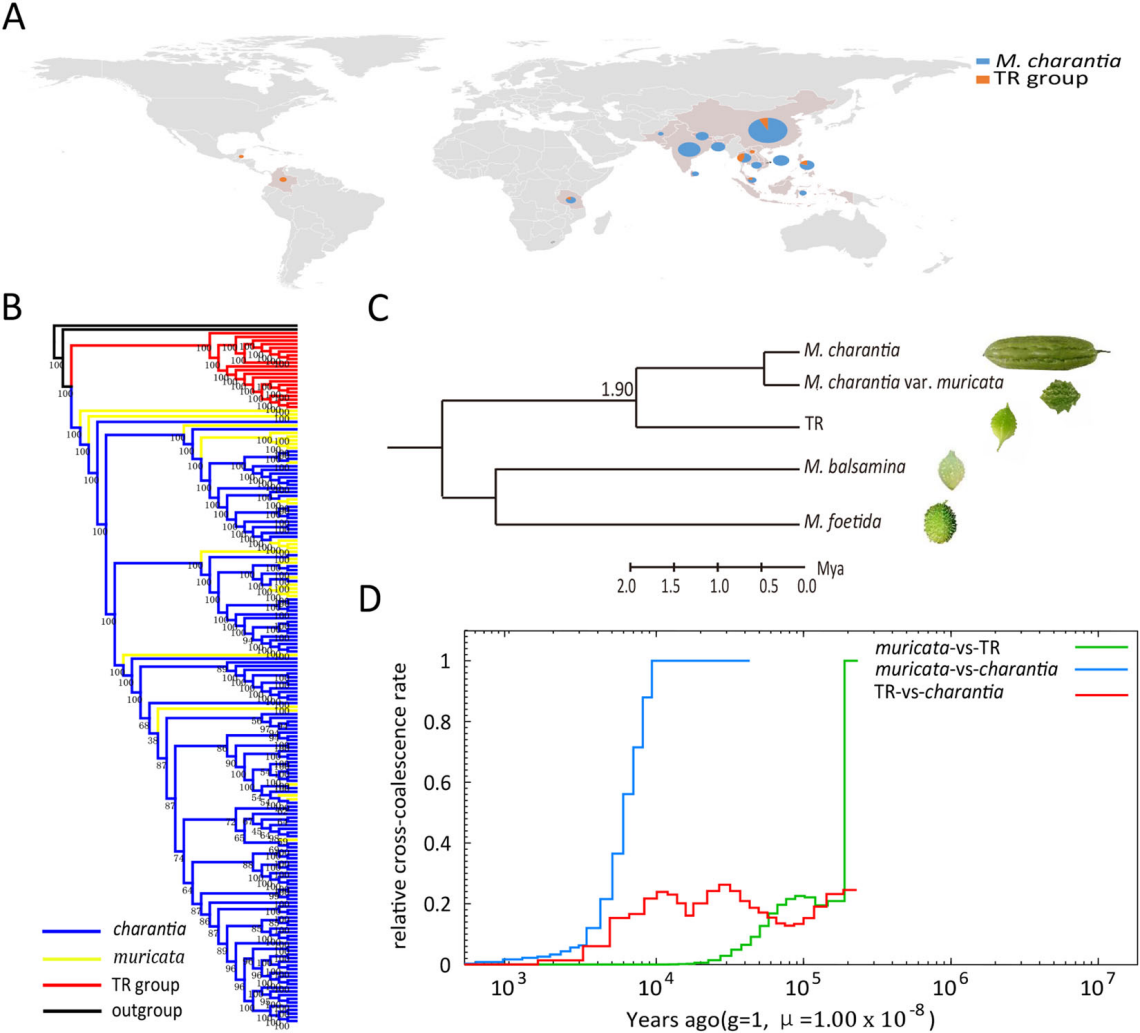
Evolution of bitter gourd. **a** The geographic distribution of the 187 bitter gourd samples included in the population analysis. **b** Neighbor-joining tree for 189 *Momordica* samples with different taxa represented by different colors: TR (red), *muricata* (yellow), *charantia* (blue), and *M. balsamina* and *M. foetida* (black) as outgroups. **c** Phylogeny of *Momordica* with the inferred divergence time between TR and *M. charantia* (Supplementary Fig. S10). **d** MSMC plots of inferred genetic divergence times (colored lines) among TR, *muricata*, and *charantia*. The split between the two genomes is defined as the time when the cross-coalescence rate drops to 50%

We resequenced 189 *Momordica* accessions selected from a panel of 212 accessions collected from around the world^68^ (Fig. 2a and Supplementary Table S27). Among these accessions, one *M. balsamina* and one *M. foetida* sample were designated as the outgroup. We generated ~8.2 billion clean paired-end reads (~1.0 trillion base pairs of sequences), with an average GC ratio of 37.0% and Q20 of 92.1% (Supplementary Table S27). After aligning these clean reads to the Dali-11 genome, the mapping rate ranged from 88.2% to 98.8%, and the average depth ranged from 8.8 to 37.8 among different samples (Supplementary Table S28). Furthermore, we identified a total of 14,450,193 SNPs and 1,588,578 InDels (shorter than 50 bp; Supplementary Tables S29 and S30). Next, we aligned the clean reads to the TR genome and identified 12,170,007 SNPs and 1,572,660 InDels (Supplementary Tables S31–S33). To analyze the evolutionary history of bitter gourd, we conducted a phylogenetic analysis using the separated and combined whole-genome SNPs called from the Dali-11 and TR assemblies and rooted the tree with *M. foetida*. Interestingly, we found that the 21 small-fruited samples showing a similar morphology to TR formed a distinct clade (Fig. 2b and Supplementary Figs. S8 and S9, Supplementary Tables S34–S36), suggesting that they are a distinct monophyletic group (temporarily designated the TR group) that originated independently.

The divergence time between the *M. charantia* and TR group was estimated to be ~1.9 Mya (Fig. 2c and Supplementary Fig. S10), which is much longer than the history of human domestication (~10,000 years)^69^. We then conducted multiple sequential Markovian coalescent (MSMC) analysis with the Dali-11 and TR genome sequences and THMC155 (*muricata*) resequencing data. The results showed that Dali-11 diverged from TR >200,000 years ago, and that Dali-11 diverged from THMC155 ~6000 years ago (Fig. 2d). We further identified 6,595,112 SNPs in the 166 *M*. *charantia* samples and 6,098,414 SNPs in the 21 TR-group samples (Table 2 and Supplementary Table S37). The nucleotide diversity values (*θπ*, *θw*) for the TR group samples (*θπ* = 6.59 × 10^−3^, *θw* = 5.37 × 10^−3^) were significantly higher than those for the *M. charantia* population (*θπ* = 1.76 × 10^−3^, *θw* = 3.88× 10^−3^; Table 2 and Supplementary Fig. S11). The fixation index value (*F*_ST_) between the two populations reached 0.85 (Supplementary Fig. S11 and Supplementary Table S38). We found that TR group samples (Tajima’s *D* = 0.79) may lack rare alleles or that the group may be under balancing selection. In comparison, the *M. charantia* population (Tajima’s *D* = −1.71) may harbor an excess of rare alleles or have recently undergone a population expansion (Table 2)^70^. Moreover, *M. charantia* exhibited a higher average d*N*/d*S* ratio in genic regions (1.24) compared to the TR group (1.05; Table 2). We found considerable differences between the genetic diversity of the TR group and *M. charantia* populations. The TR group diverged from *M*. *charantia* before human domestication. Overall, these results suggest that the TR group may be a new species or subspecies independent of *M. charantia*.

**Table 2:** General information on genetic variation in the bitter gourd genome

Whole genome	Sample no.	SNP no.	*θπ*(10^−3^)	*θw*(10^−3^)	Tajima’s *D*
*M. charantia*					
South Asia	50	3,311,877	1.94	2.42	−0.79
Southeast Asia	49	5,036,642	1.92	3.73	−1.61
China	62	1,764,264	0.69	1.23	−1.45
Tanzania	5	880,137	–	–	–
Total	166	6,595,112	1.76	3.88	−1.71
TR group	21	6,098,414	6.59	5.37	0.79

Genic regions	Sample no.	SNP no.	*θπ*(10^−3^)	*θw*(10^−3^)	Tajima’s *D*	Average nonsyn SNPs	Average syn SNPs	Average d*N*/d*S*
*M. charantia*								
South Asia	50	506,143	0.81	1.24	−1.18	8358	6608	1.27
Southeast Asia	49	774,674	0.91	1.92	−1.80	6381	5445	1.20
China	62	298,347	0.35	0.70	−1.67	3208	2566	1.25
Tanzania	5	131,308	–	–	–	–	–	–
Total	166	1,133,465	0.80	2.25	−2.02	5877	4777	1.24
TR group	21	847,989	2.82	2.51	0.46	52,571	49,998	1.05

### Geographic diversity of *M. charantia*

**Fig. 3:**
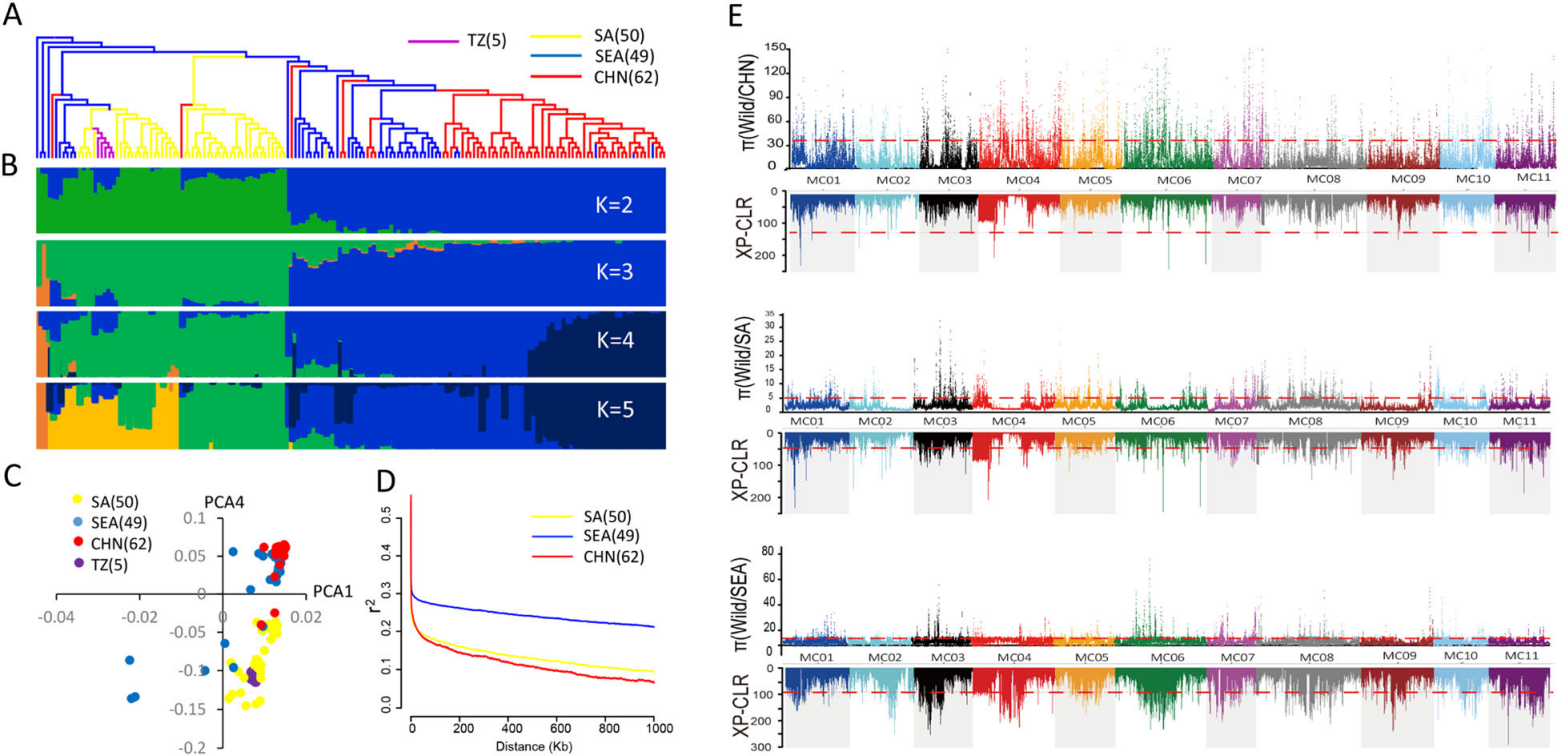
Population analysis of *M*. *charantia*. **a** The neighbor-joining tree of 166 *M. charantia* samples. **b** Model-based clustering analysis with cluster numbers of two to five. **c** Principal component analysis of *M*. *charantia* from different regions. **d** Decay of LD measured by r^2^ in the four geographic groups. **e** Whole-genome screening of domestication regions in the populations of CHN30, SA30, and SEA30. SA South Asia, SEA Southeast Asia, CHN China, TZ Tanzania. The horizontal red dashed lines indicate the threshold defining the top 5% of *π* values (above the lines) and XP-CLR scores (below the lines)

Based on a neighbor-joining tree, we found that all 30 samples of *muricata* were nested within the cultivated *M*. *charantia* clade and that many were basal to a cluster of *M*. *charantia* (Fig. 2b), supporting the aforementioned conclusion that *muricata* is the wild progenitor of *M. charantia*. In addition, the 166 *M. charantia* germplasms can be separated into four geographically differentiated gene pools: South Asia, Southeast Asia, China, and Tanzania (Fig. 3a). This geographic division was illustrated by both population stratification and principal component analysis (PCA) (Fig. 3b, c and Supplementary Table S39). The germplasms from South Asia and China were more differentiated, and the germplasms from Southeast Asia were relatively mixed and exhibited genetic heterogeneity (Fig. 3b, c). In total, we identified 3,311,877, 5,036,642, 1,764,264, and 880,137 SNPs in the South Asia, Southeast Asia, China, and Tanzania groups, respectively (Table 2). The South Asia group exhibited the highest genetic diversity, with a *θπ* value of 1.94 × 10^−3^ (Table 2). Tajima’s *D* in the South Asia group (–0.79) was higher than those in the Southeast Asia (–1.61) and China groups (–1.45), suggesting lower genetic diversity in the last two groups (Table 2). The different geographic groups exhibited variable LD decay values, among which the Southeast Asian population presented the highest (42.6 kb), followed by the Chinese (1.5 kb) and South Asian (0.7 kb) populations (Fig. 3d). These findings support the hypothesis that the domestication of *M. charantia* in Asia was driven in South Asia^71^. Furthermore, we found that the 30 *muricata* samples were distributed across South Asia, Southeast Asia, and China, suggesting either that wild and cultivated bitter gourd dispersed together or that there were multiple domestication events. To identify the genetic regions under selection, we selected 30 large-fruited *M. charantia* samples from each of South Asia, Southeast Asia, and China (the selected groups are referred to as SA30, SEA30, and CHN30, respectively) and calculated the diversity ratios and the XP-CLR values between the geographic groups and the *muricata* population (Wild30). Combining the top 5% of *θπ* and XP-CLR outliers, we identified 6854, 9794, and 7052 selected regions in SA30, SEA30, and CHN30 populations, comprising 710, 412, and 290 genes, respectively (Fig. 3e and Supplementary Figs. S12–S14, Supplementary Tables S40 and S41). Many of these domestication genes were enriched in various metabolic processes (Supplementary Tables S42–S44). These candidate domestication genes will provide the foundation for the identification of associations with key domestication traits.

### Comparative analysis of cucurbitane triterpenoid biosynthesis genes

**Fig. 4:**
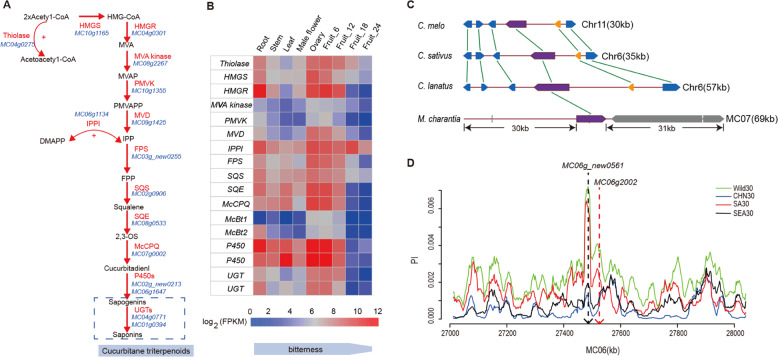
Genes involved in the cucurbitane triterpenoid pathway. **a** The cucurbitane triterpenoid biosynthesis pathway in bitter gourd; the red text denotes the enzymes along with the corresponding predicted enzyme-encoding genes (blue). **b** Heat map of gene expression for cucurbitane triterpenoid biosynthesis candidate genes. Log_2_-scaled fragments per kilobase per million (FPKM) values are shown, ranging from low (blue) to high (red) expression. Fruit-6, Fruit-12, Fruit-18, and Fruit-24 represent four developmental stages (6, 12, 18, and 24 days after pollination). The “bitterness” bar indicates that the bitter gourd root, stem, leaf, male flower, ovary, fruit-6, and fruit-12 tissues are bitter, while bitterness decreases from fruit-18 to fruit-24. **c** The distribution of *Bi* clusters in cucurbits. *Bi* clusters are conserved in *C. sativus*, *C. melo*, and *C. lanatus* but lost in *M. charantia*. The conserved gene clusters span regions of 30–69 kb consisting of six genes, purple: *OSCs*; blue: *P450s*; gold: *ACTs*; gray: unrelated genes. **d** Distribution of nucleotide diversity (*π*) at the *MC06g_new0561* (*McBt1*) and *MC06g2002* (*McBt2*) loci in four populations of bitter gourd. SA South Asia, SEA Southeast Asia, CHN China

Cucurbitane triterpenoids are the major bitter substances in various cucurbit vegetables, and they are synthesized through the mevalonate pathway^72^. Cucurbitadienol synthase (CPQ)^73^ catalyzes the conversion of 2,3-oxidosqualene (2,3-OS) to cucurbitadienol, resulting in the basic skeletal structure of cucurbitane triterpenoid (Fig. 4a). Then, the cucurbitadienol skeleton is further modified by tailoring enzymes, mainly cytochrome P450s (P450s) and UDP-glycosyltransferases (UGTs), to produce diverse cucurbitane triterpenoids. The *CPQ* orthologs in cucumber and watermelon are *Bi*^74^ and *CcCDS2*/*cla007080*, respectively^75^. We identified *MC07g0002* as the closest homolog of *CPQ* in bitter gourd, and the function of this gene in cyclizing 2,3-oxidosqualene to generate cucurbitadienol in yeast has recently been validated^76^. The *CPQ* phylogenetic tree showed that *MC07g0002* clustered with *Siraitia grosvenorii CPQ* (*SgCPQ*), forming a group separated from the orthologs in *C. pepo*, *C. sativus*, *C. melo*, and *C. lanatus* (Supplementary Fig. S15). We found that *MC07g0002* expression was not tissue specific and that the expression level was positively correlated with the bitterness of the tissues, including different developmental stages of fruit (Fig. 4b). Other cucurbits have a conserved *Bi* cluster responsible for the biosynthesis of cucurbitacin C, B, and E in the same genomic region^74,77^. We used *MC07g0002* as bait to search for other coexpressed genes with predicted functions. Interestingly, the main putative cucurbitane triterpenoid biosynthesis genes, including *McCPQ* (*MC07g0002*), two *P450s* (*MC02g_new0213* and *MC06g1647*), and two *UGTs* (*MC04g0771* and *MC01g0394*), were not genetically linked to *McCPQ* in the bitter gourd genome (Fig. 4c and Supplementary Figs. S16–S19). This is similar to the mogroside pathway genes found in the *S. grosvenorii* genome^78^.

Two homologs of the bHLH transcription factors *Bl* and *Bt* regulate the expression of cucurbitane triterpenoid biosynthesis in cucumber leaves and fruits^74^, respectively. We identified *MC06g_new0561* and *MC06g2002* as orthologs of these genes in bitter gourd (Supplementary Fig. S20 and Supplementary Table S45). Both *MC06g_new0561* (*McBt1*) and *MC06g2002* (*McBt2*) were moderately expressed from the ovary to the fruit-12 period and showed weaker expression from the fruit-18 to fruit-24 periods; their expression pattern was highly similar to the expression of *MC07g0002* in bitter gourd fruit (Fig. 4b). We also found that *MC06g2002* exhibited a higher expression level in bitter gourd roots compared with *MC06g_new0561* (Fig. 4b). As a result of domestication, the other three cucurbits, cucumber, melon, and watermelon, underwent a convergent reduction in the genetic diversity of *Bl* and *Bt*^77^. Interestingly, we did not observe obvious reductions in the genetic diversity of *McBt1* and *McBt2* in the three cultivated geographic populations of bitter gourd, suggesting that there may be a weak signature of artificial selection in bitter gourd around genes regulating bitterness (Fig. 4d). In addition, we found that the region of *McCPQ* (*MC07g0002*) presented the same haplotype in 90.6% of the *M. charantia* samples (including both wild and cultivated bitter gourds) (Supplementary Fig. S21). Thus, bitterness has not been intensively selected among modern bitter gourd cultivars.

## Discussion

Bitter gourd is widely consumed in many Asian countries and is used in dietary interventions for diabetes. Here, we report high-quality genome sequences for bitter gourd. Compared to a previous ab initio prediction of genes using the OHB3-1 line^15^, we provide a more confident gene set using the Dali-11 genome. Bitter gourd represents an early-branching clade of the family Cucurbitaceae^45,79^, and the genome sequences offer an opportunity to investigate the genomic and biological characteristics of early cucurbits.

By resequencing diverse bitter gourd samples, we gained valuable insights into the genetic diversity, taxonomy, and domestication of bitter gourd. In addition, we provide further evidence that southern Asia is a domestication center of bitter gourd. The differentiation of the 21 TR group samples from *M. charantia* was firmly supported by our molecular phylogenetic analyses, which were consistent with fruit and seed morphology. We deduced that the 21 TR accessions may belong to a new species or subspecies independent of *M. charantia*, or the results could support the previously reported *M. charantia* ssp*. macroloba*^80^. The genetic variation between the two species or subspecies can contribute to the utilization of bitter gourd germplasm resources through inter-specific or inter-subspecific crosses to yield improved cultivars in the future. Our findings regarding the geographic diversity and domestication of *M. charantia* lay the groundwork for future genetic improvement in bitter gourd.

In particular, the *Bi* gene cluster, which regulates cucurbitane triterpenoid biosynthesis in other popular cucurbit crops, appears to have evolved after the divergence of bitter gourd. The clustering of genes at the *Bi* locus leads to co-inheritance, co-expression and co-regulation of genes^81,82^ and may have been driven by intense selection, possibly making this an important locus for rapid responses to stresses^83^. The lack of co-inheritance of biosynthetic genes and the weak selection for regulatory genes indicate that cucurbitane triterpenoids may play a different role in the response to environments, which may also underlie the bitterness of the bitter gourd fruit. Different tailoring genes, such as *P450s* and *UGTs*, can influence the properties of the final structures of cucurbitane triterpenoids^77,81^, which may also contribute to the bitterness of bitter gourd compared with other cucurbits. Future functional validation will help to clarify these differences.

## Supplementary information

Supporting information S1 figures

Supporting information S2 tables

Supporting information S3 tables

## Data Availability

The bitter gourd genome data have been deposited in the CNGB Nucleotide Sequence Archive (CNSA) (https://db.cngb.org/cnsa/home/; accession: CNP0000016). The RAD data have been deposited at CNGB (https://db.cngb.org/cnsa/; accession: CNP0000012) and the European Nucleotide Archive (ENA) (https://www.ebi.ac.uk/ena/data/view/PRJEB23602).
