# Supplementary material for: Whole-genome sequencing provides insights into the genetic diversity and domestication of bitter gourd (Momordica spp.)
Source: Hortic Res. 2020 Jun 1;7:85. doi: 10.1038/s41438-020-0305-5 (PMC7261802; doi:10.1038/s41438-020-0305-5)
Supplement: Supplementary file 1 — Supporting information S1 figures [file 41438_2020_305_MOESM1_ESM.pdf]

Supplementary Figures

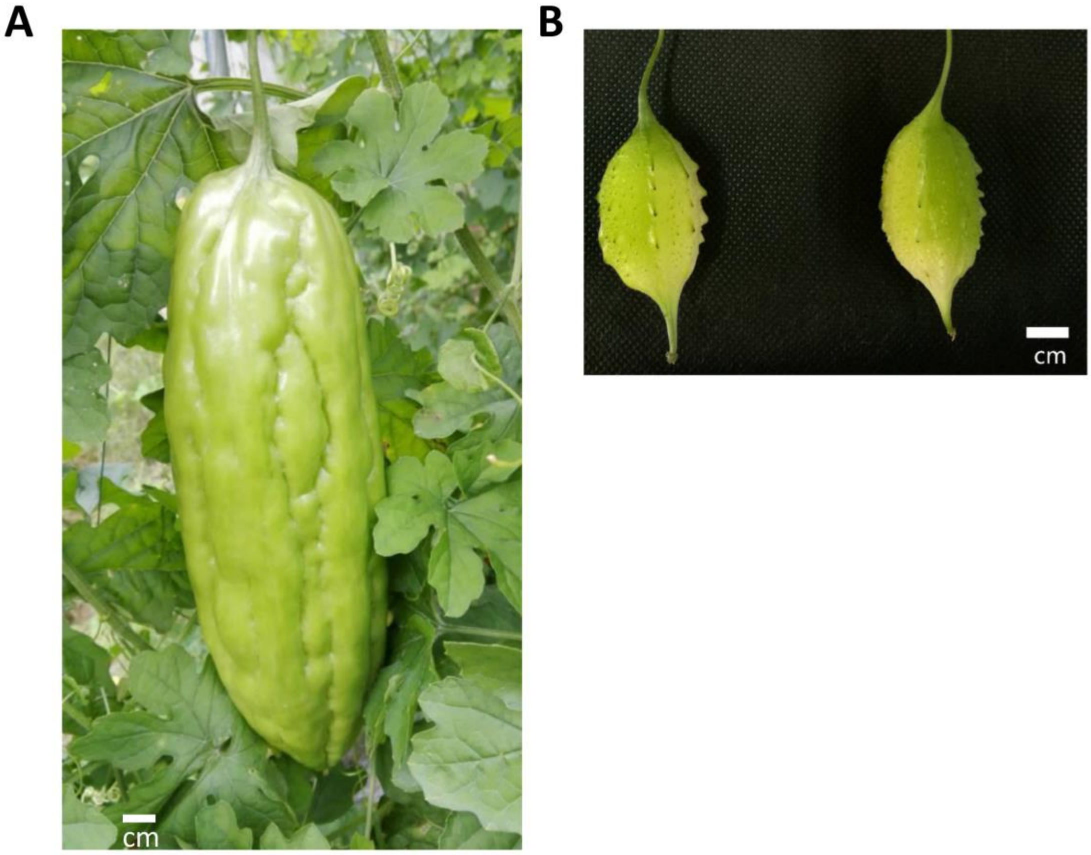

Figure S1. Fruit pictures of the two bitter gourd samples used for *de novo* sequencing in this study. (**A**) Dali-11, (**B**) TR. Size bar, 1 cm.

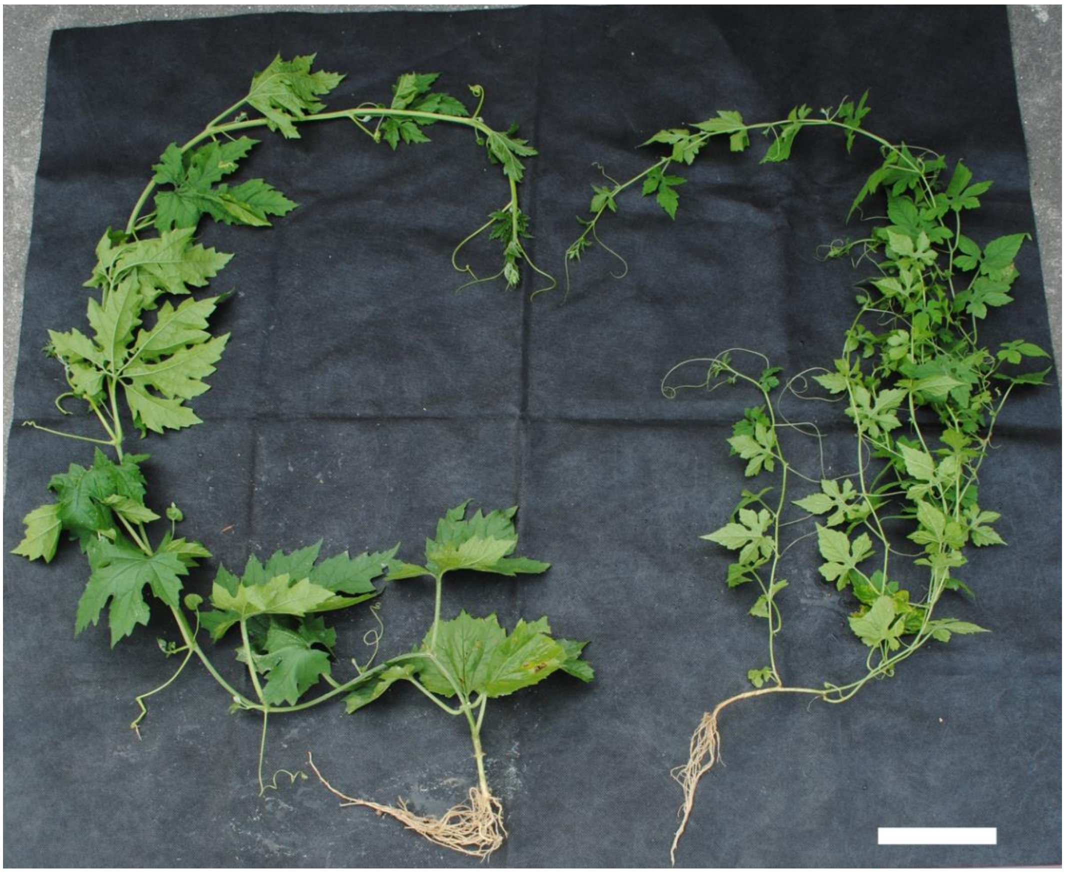

Figure S2. The plants of *M. charantia* Dali-11 (left) and TR (right) (30 days after planting).

White scale bar represents 10 cm.

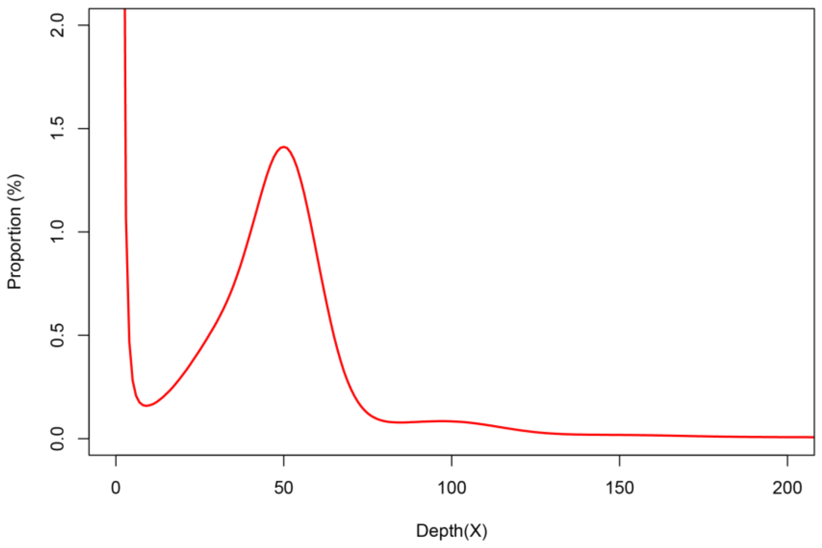

Figure S3. 17-mer frequency distribution of sequencing reads for Dali-11. The x-axis is depth (×); the y-axis is the proportion, which represents the frequency at that depth divide by the total frequency of all the depth. A total of 14,993,541,395 17-mers were obtained and the peak depth was 50.

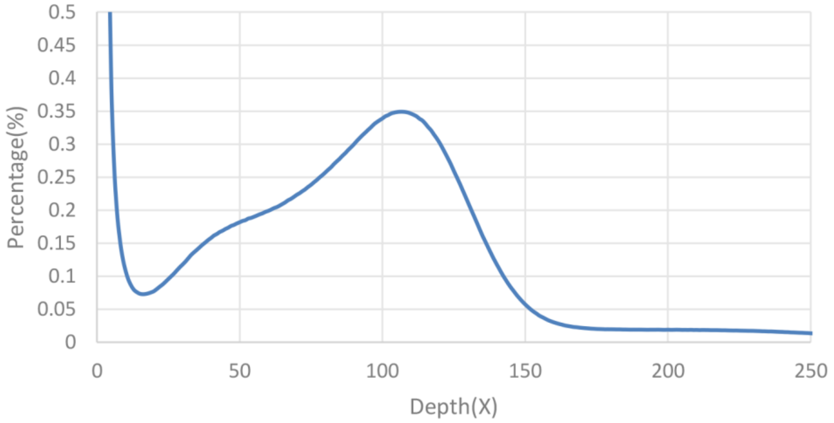

Figure S4. 17-mer frequency distribution of sequencing reads for TR. A total of 32,178,510,001 17-mers were obtained and the peak depth was 107.

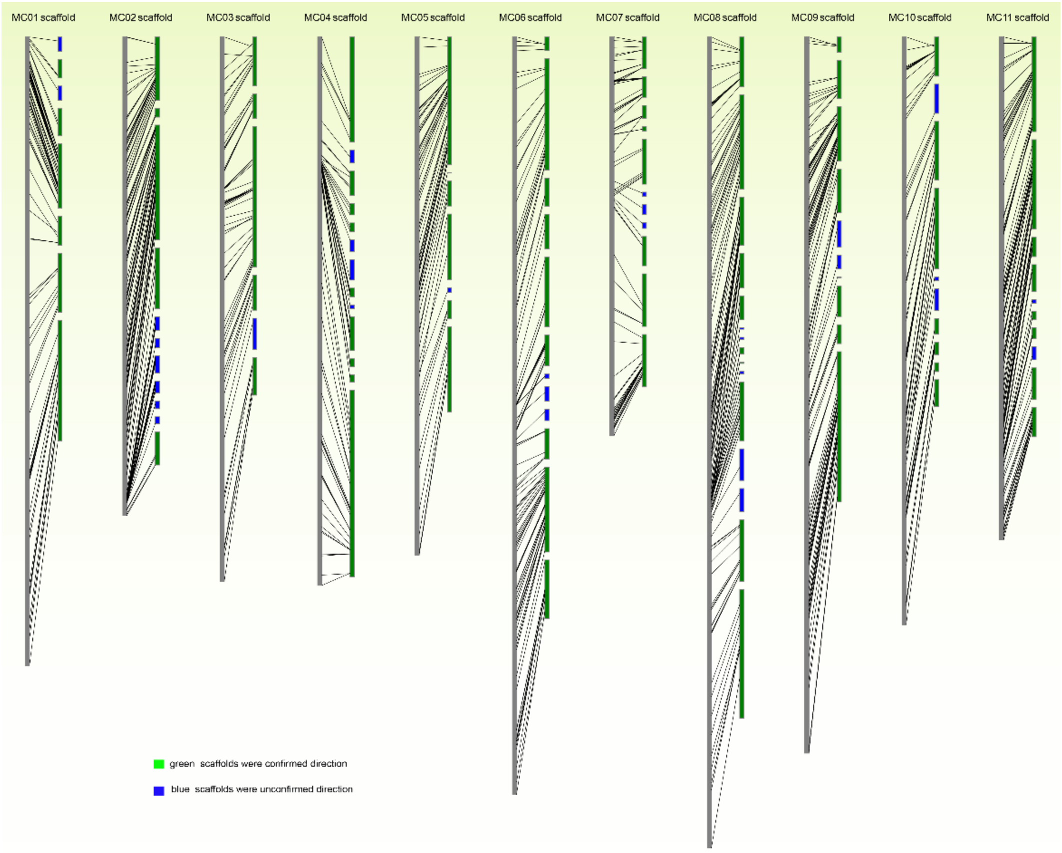

Figure S5. Bitter gourd pseudochromosomes construction based on the RAD map. Assembled scaffolds were anchored on the 11 linkage groups using 1,009 high-quality markers. Oriented scaffolds are represented in green and non-oriented scaffolds are in blue.

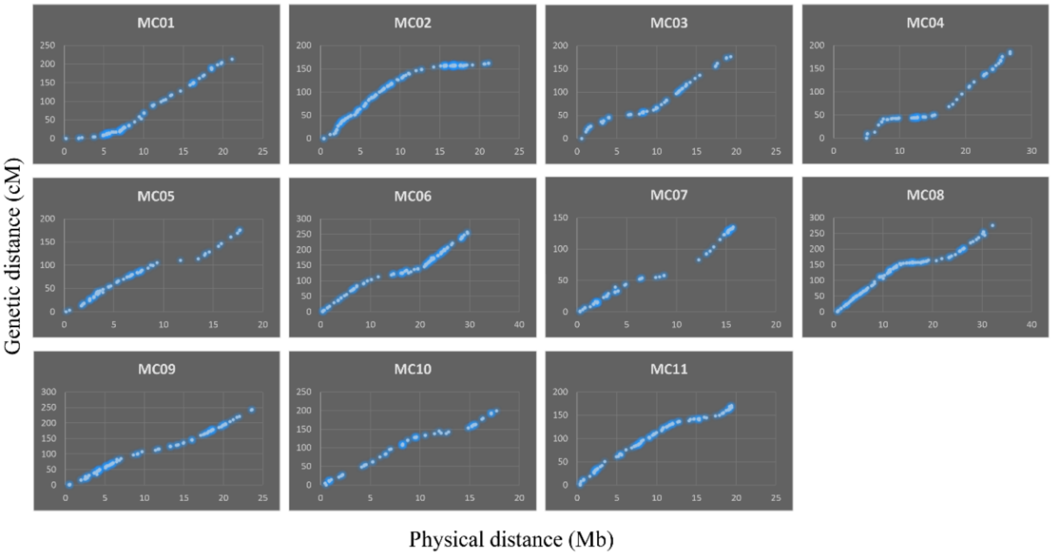

Figure S6. Comparison of genetic and physical distance of the 11 bitter gourd pseudochromosomes.

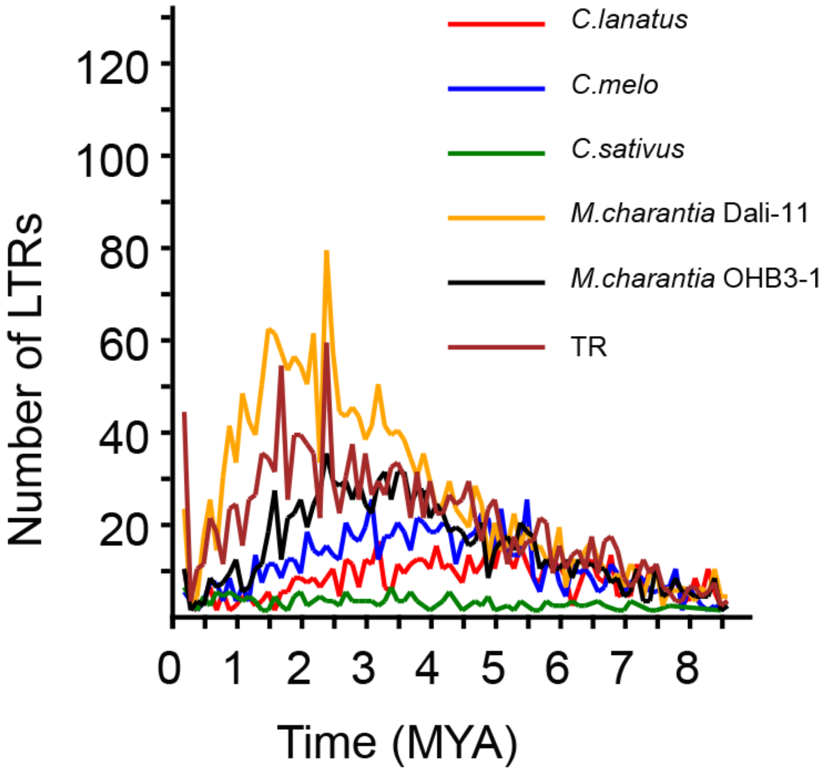

Figure S7. Distribution of LTR insertion time in cucurbits. MYA: million years ago.

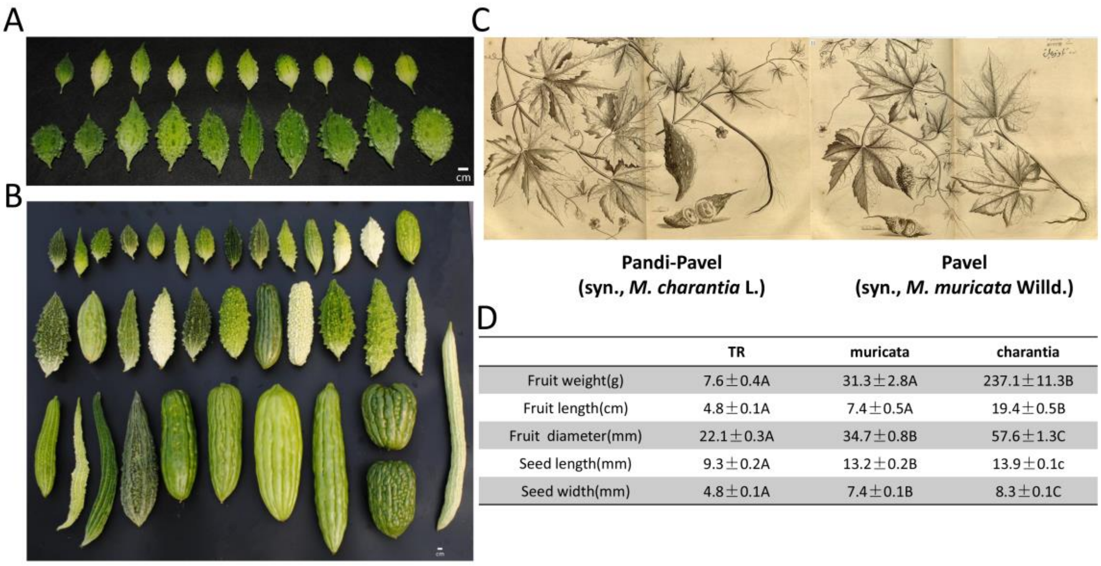

	TR	muricata	charantia
Fruit weight(g)	7.6±0.4A	31.3±2.8A	237.1±11.3B
Fruit length(cm)	4.8±0.1A	7.4±0.5A	19.4±0.5B
Fruit diameter(mm)	22.1±0.3A	34.7±0.8B	57.6±1.3C
Seed length(mm)	9.3±0.2A	13.2±0.2B	13.9±0.1c
Seed width(mm)	4.8±0.1A	7.4±0.1B	8.3±0.1C

Figure S8. Phenotypic variation of bitter gourd fruit. (**A**) Fruit variation of small-fruited bitter gourd: TR (top row) and *muricata* (bottom row). (**B**) Fruit variation of middle to large fruited bitter gourd (*charantia*). (**C**) The earliest record of bitter gourd illustrations from Hortus Malabaricus, “Pandi-pavel” and “Pavel” refer to *charantia* and *muricata*, respectively. (**D**) Variability in fruit and seed size. Scale bar represents 1 cm.

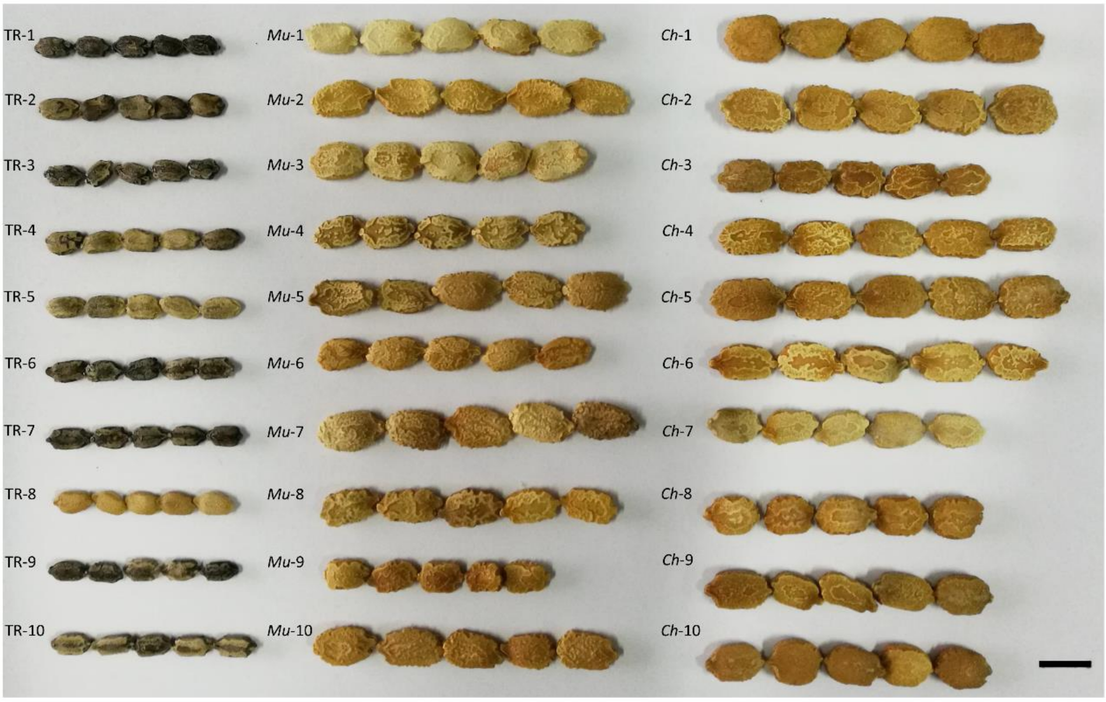

Figure S9. The seeds of TR group (TR; left), muricata (Mu; middle), and charantia (Ch; right).

Scale bar represents 1 cm.

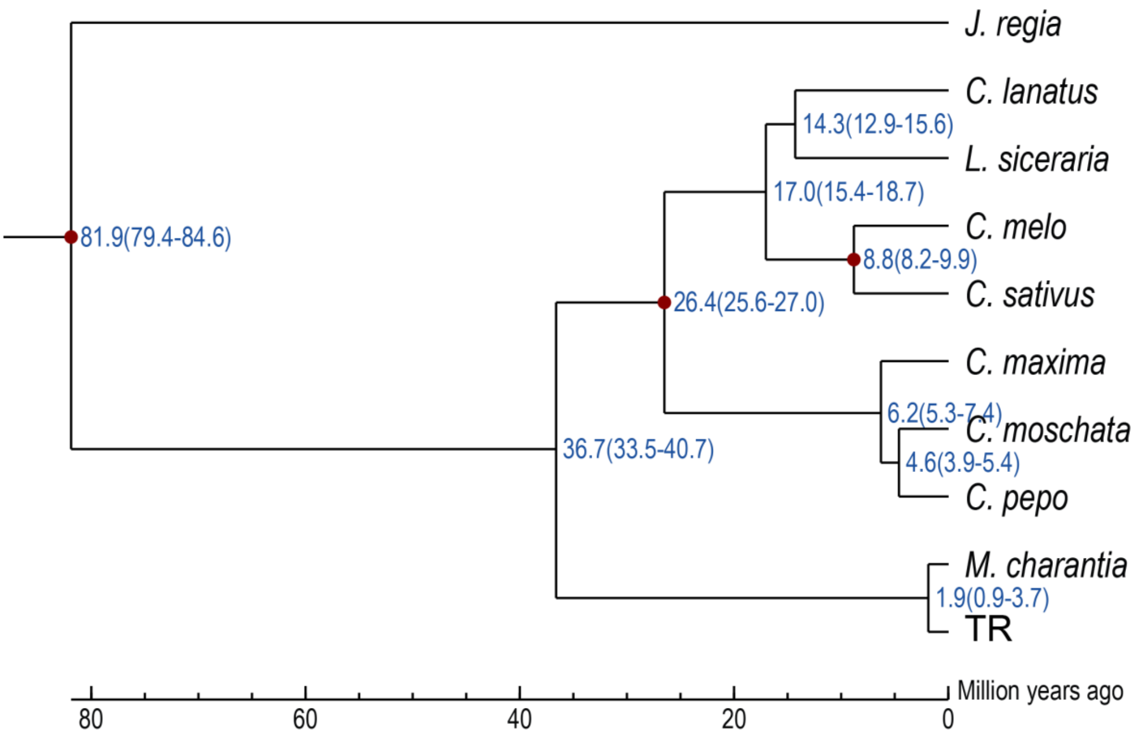

Figure S10. Phylogeny and divergent time of 10 plant species. The Bayesian tree was conducted using 2,219 shared single copy genes.

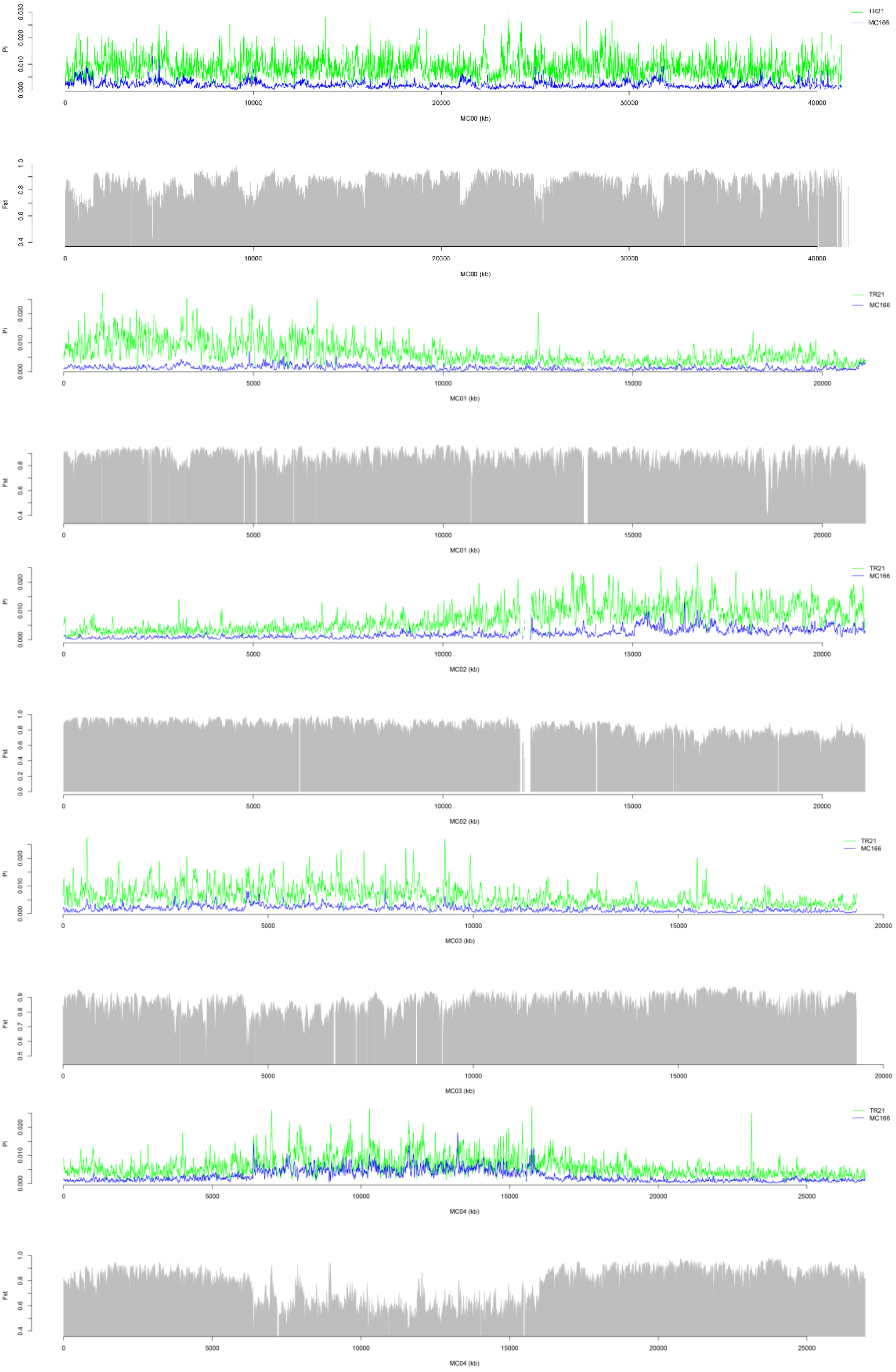
Pi
TR21
MC166
MC00 (kb)
Fst
MC00 (kb)
MC01 (kb)
MC01 (kb)
MC02 (kb)
MC02 (kb)
MC03 (kb)
MC03 (kb)
MC04 (kb)
MC04 (kb)

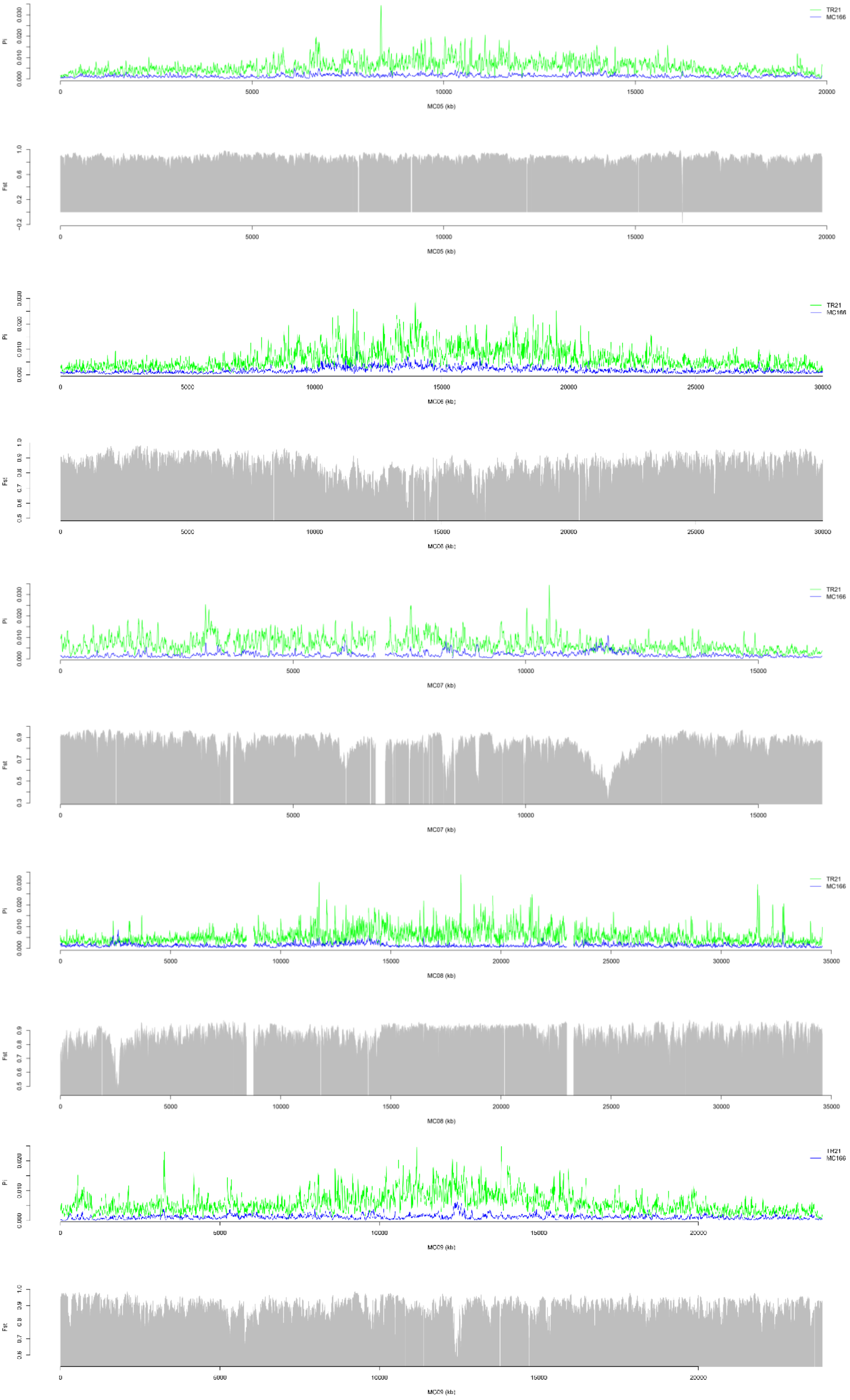
TR21
MC166
Pi
Fst
MC05 (kb)
MC06 (kb)
MC07 (kb)
MC08 (kb)
MC09 (kb)

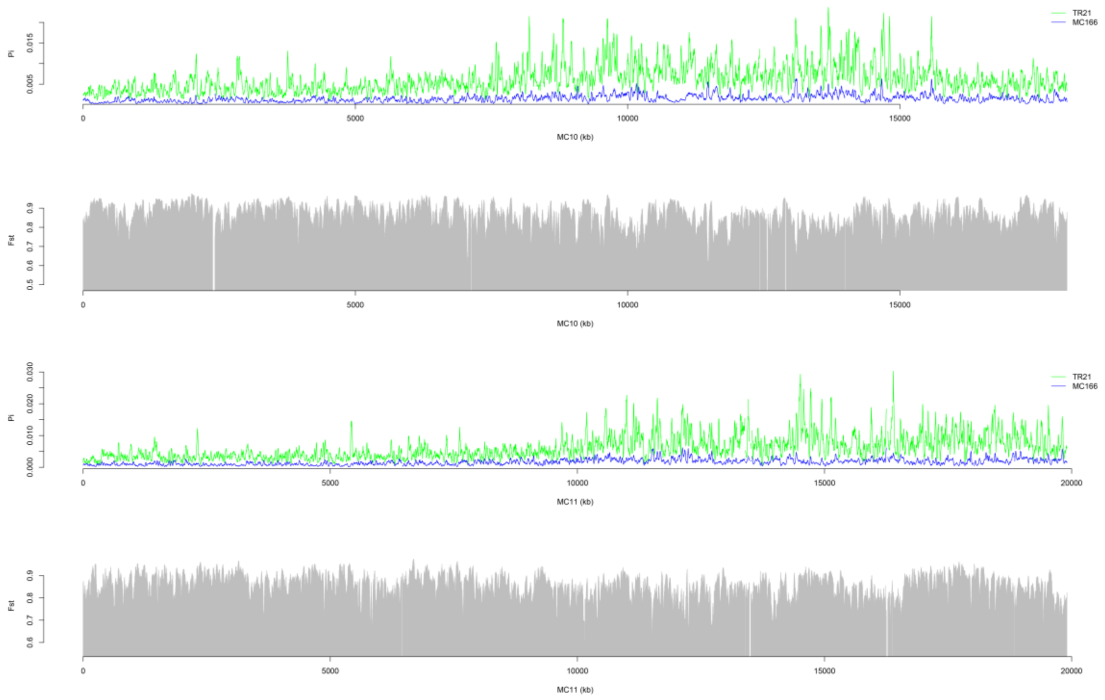

Figure S11. Diversity in bitter gourd population of 21 TR samples and 166 M. charantia samples and F_{ST} distribution between them.

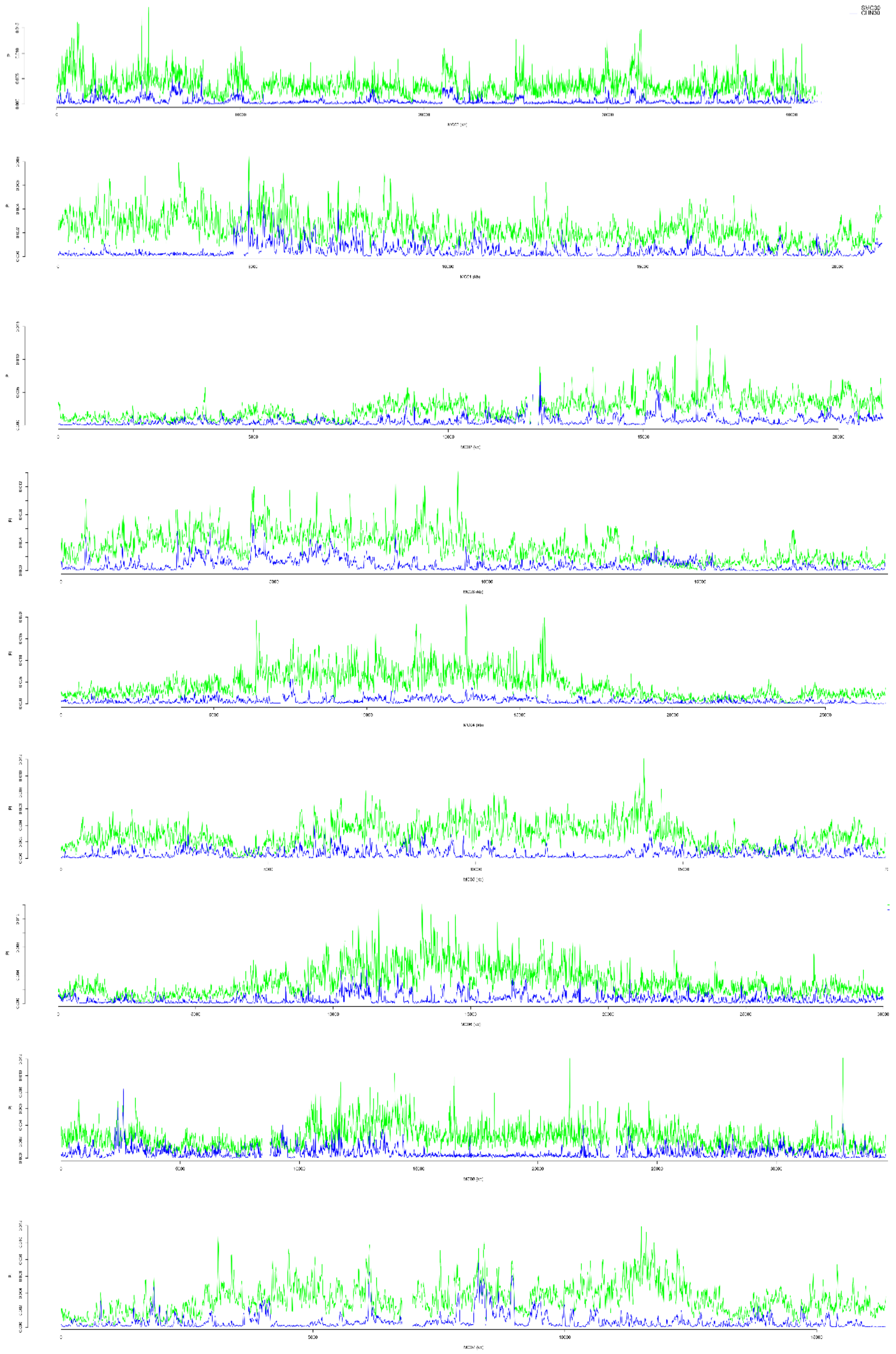
SMC30
CHN30

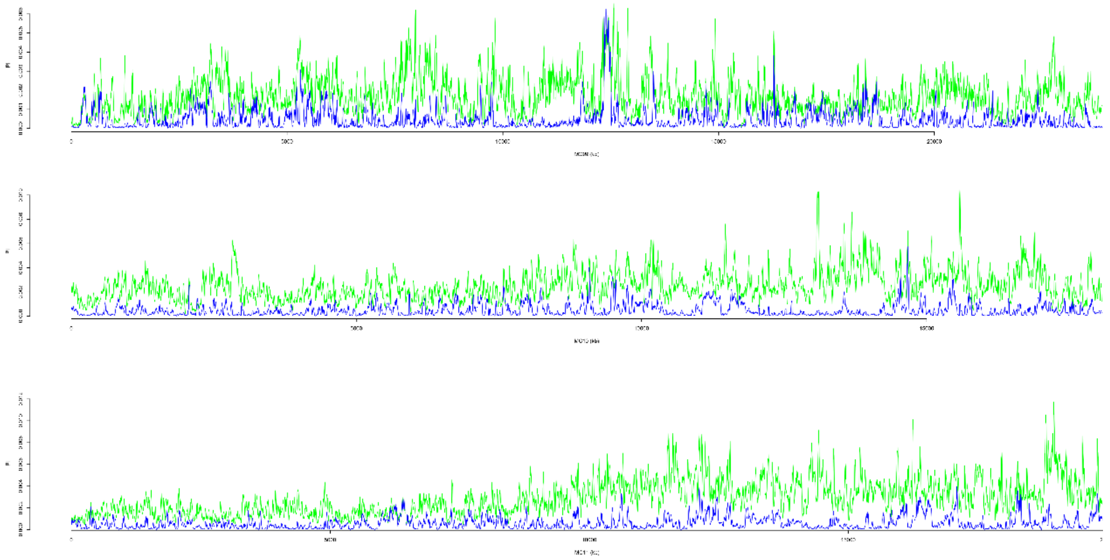

Figure S12. Diversity in bitter gourd population of Wild30 and CHN30.

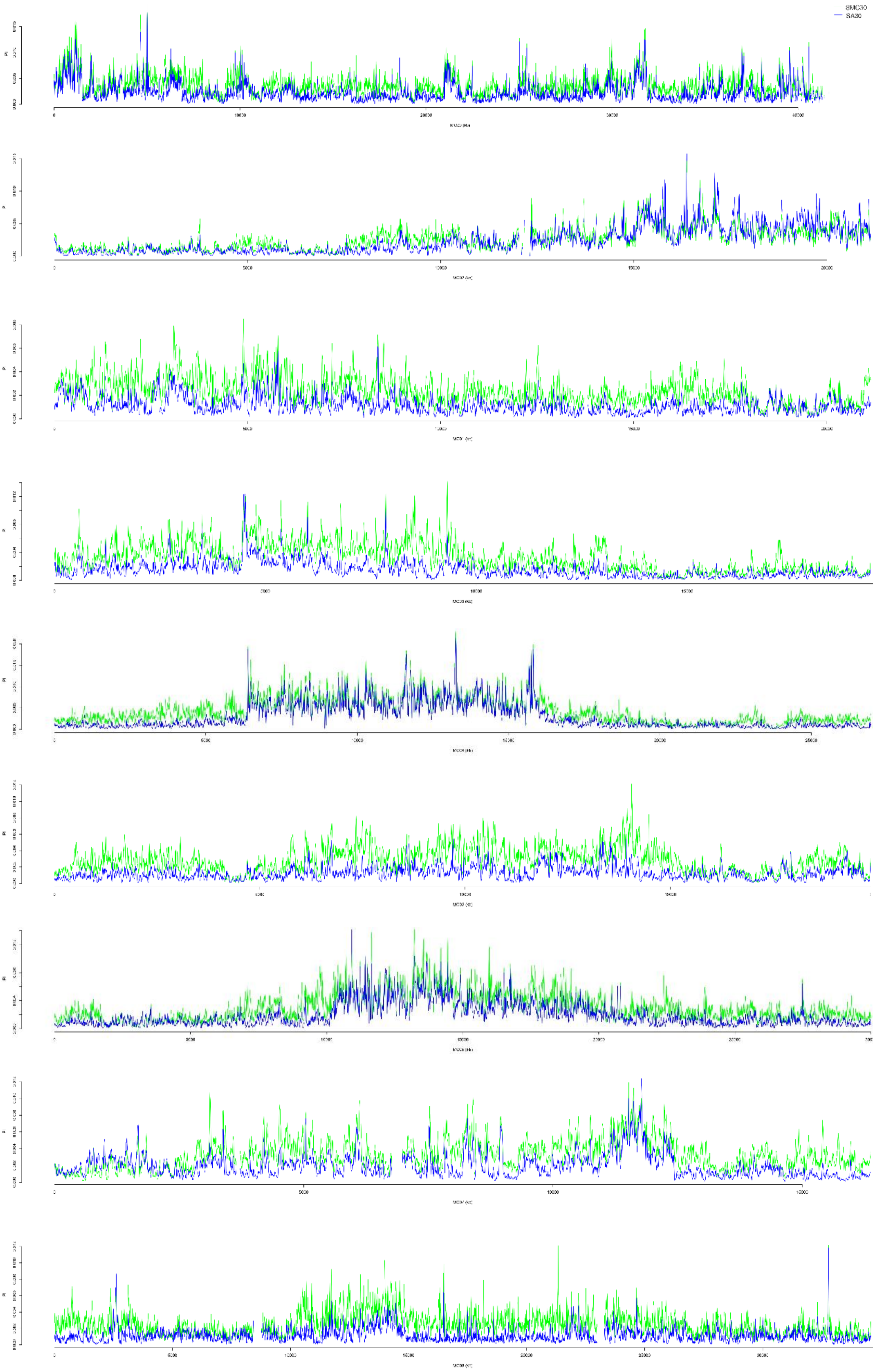
SMC30
SA30

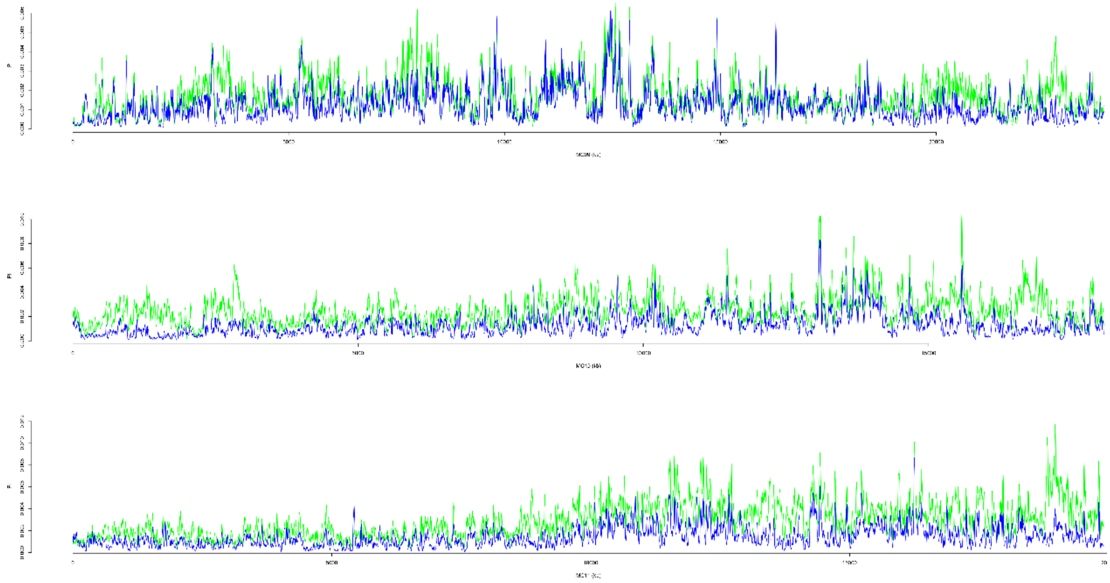

Figure S13. Diversity in bitter gourd population of Wild30 and SA30.

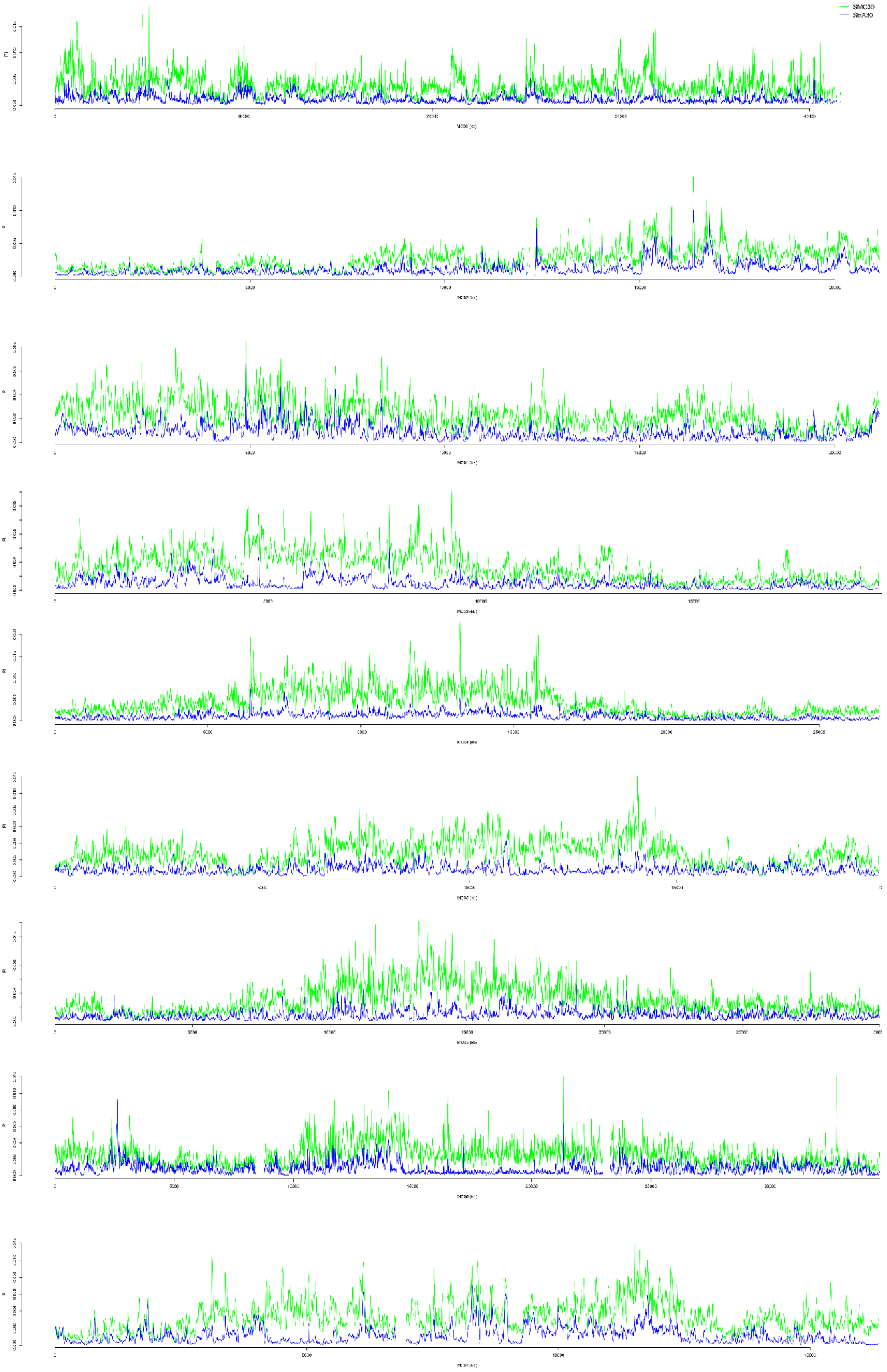
SMC30
SEA30

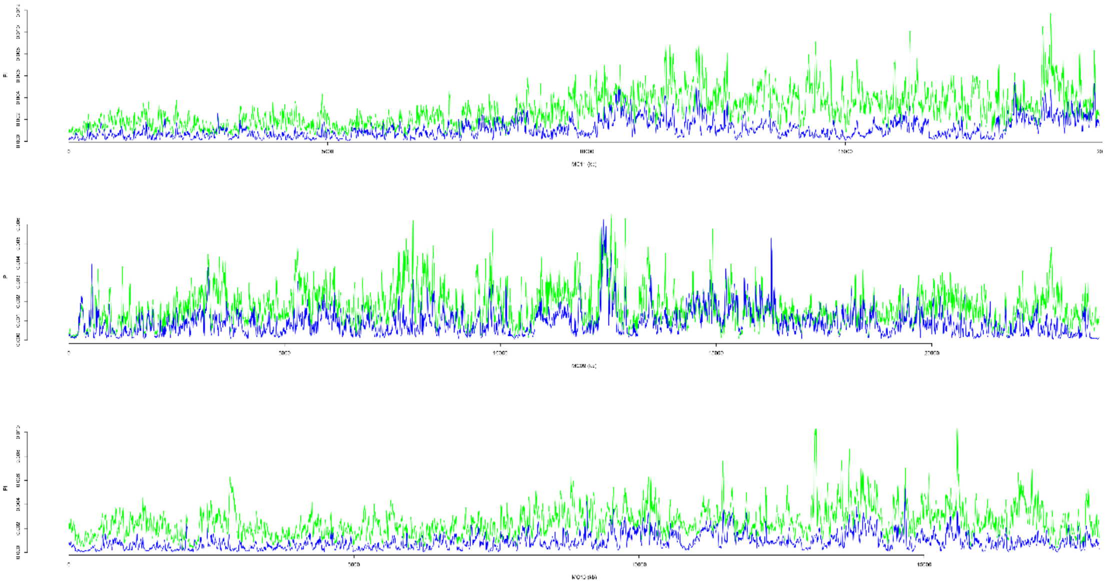

Figure S14. Diversity in bitter gourd population of Wild30 and SEA30.

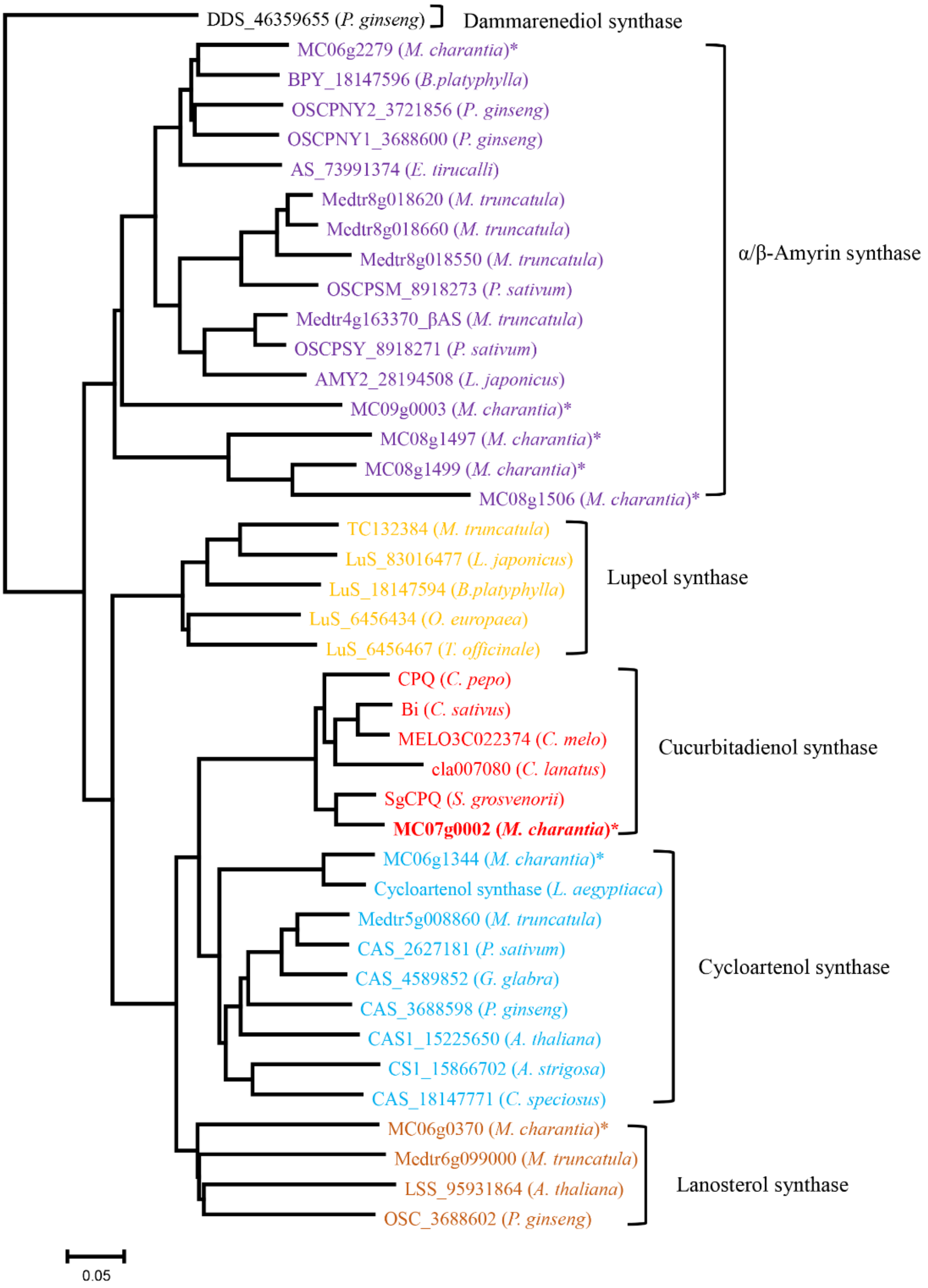

Figure S15. Phylogenetic analysis of oxidosqualene cyclases (OSCs) from *M. charantia* and other plant species. Panax ginseng dammarenediol synthase represents an outgroup taxon. *M. charantia* OSCs are indicated with asterisks and the cucurbitadienol synthase (MC07g0002) is in bold. Branches of different enzyme classes are colored: purple, α/β-Amyrin synthase; gold, lupeol synthases; red, cucurbitadienol synthase; blue, cycloartenol synthases; and orange, lanosterol synthases.

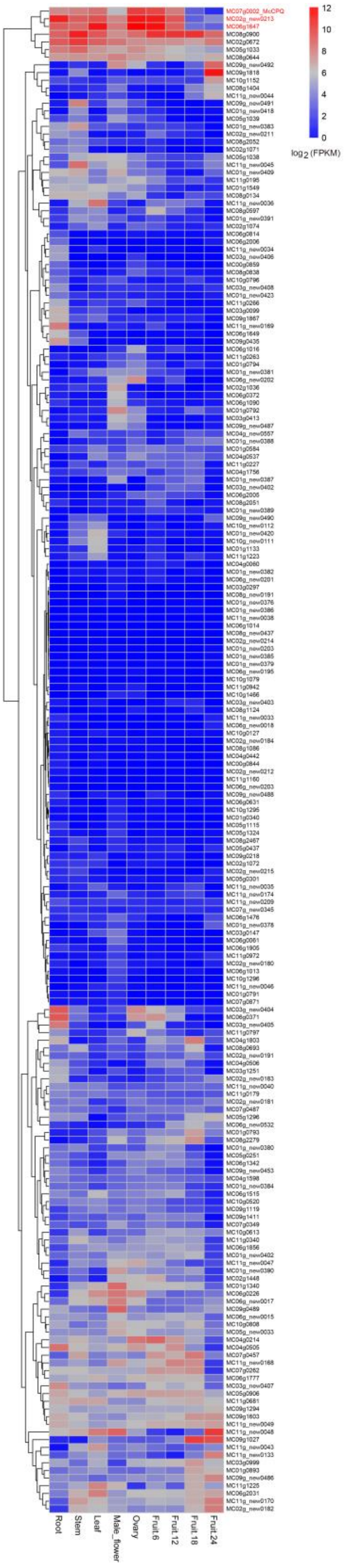

Figure S16. Hierarchical clustering analysis of gene expression patterns for *P450s*. The cucurbitadienol synthase gene *MC07g0002* was used as bait to search for 203 *P450s* with the most similar expression patterns.

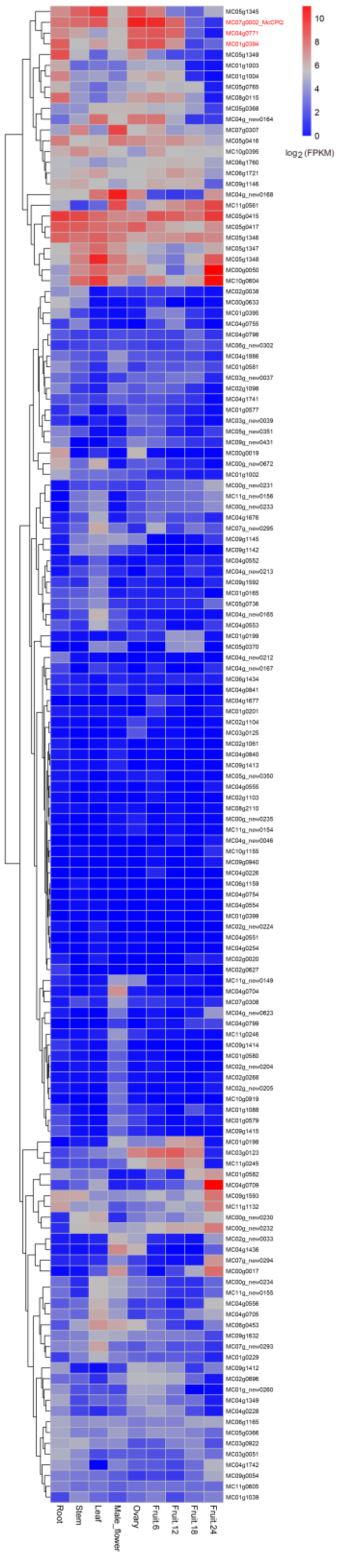

Figure S17. Hierarchical clustering analysis of gene expression patterns for *UGTs*. The cucurbitadienol synthase gene *MC07g0002* was used as bait to search for 139 *UGTs* with the most similar expression patterns.

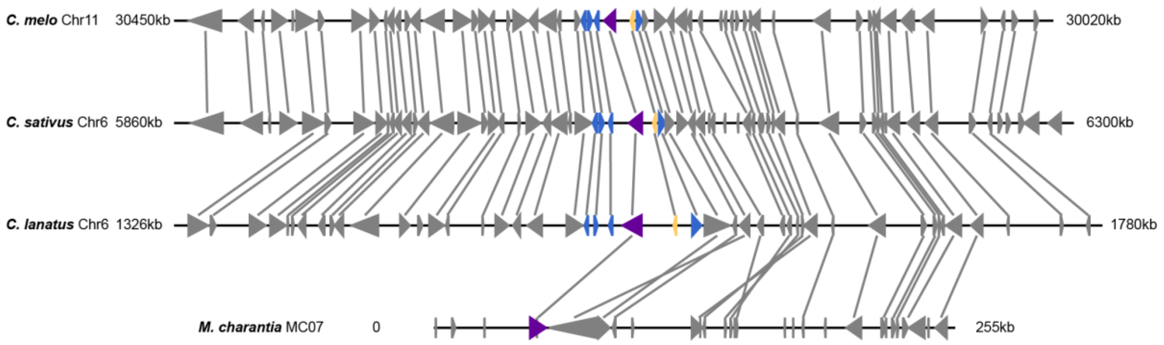

Figure S18. The synteny analysis of *Bi* clusters in cucurbits. purple: *OSCs*; blue: *P450s*; gold: *ACTs*; gray: unrelated genes.

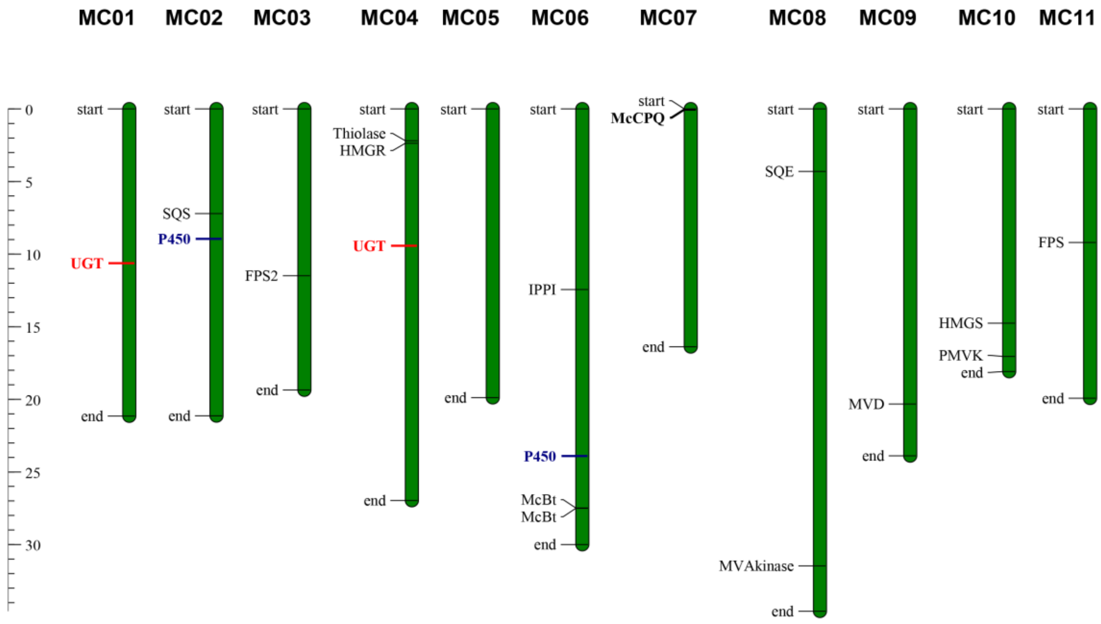

Figure S19. Map positions of genes potentially involved in cucurbitane triterpenoid pathway on 11 pseudochromosomes of *M. charantia*.

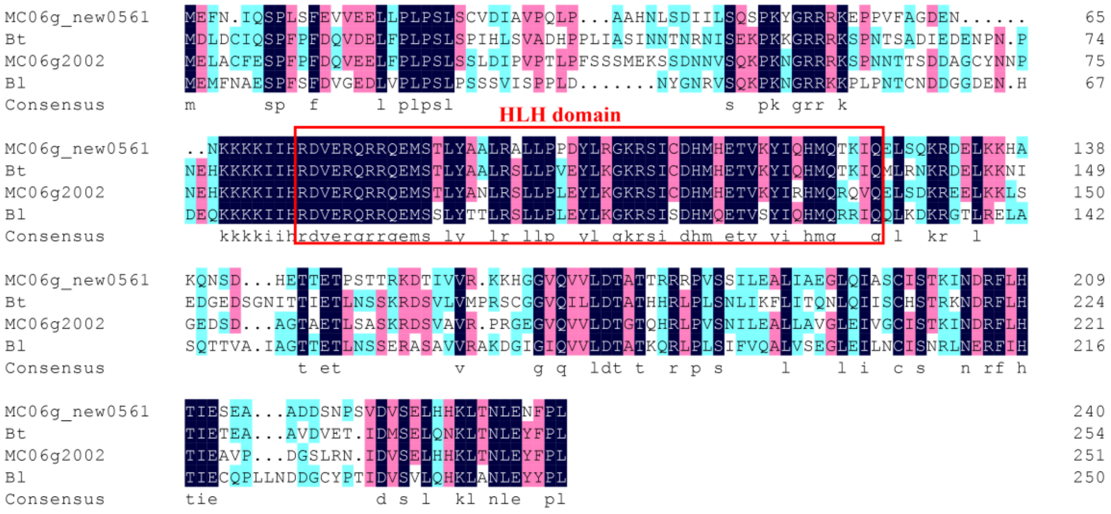

Figure S20. A consensus alignment for three bHLH transcription factor sequences from *C. sativus* (Bt and Bl) and *M. charantia* (MC06g2002 and MC06g_new0561).

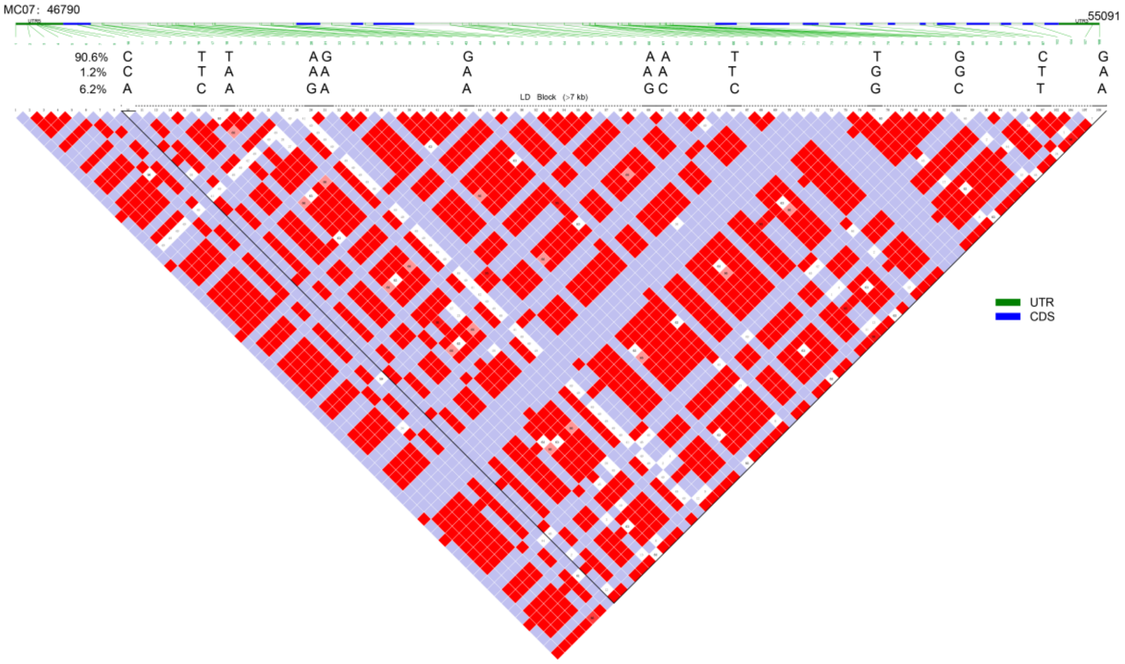

Figure S21. LD block in *McCPQ* (*MC07g0002*) among 166 *M. charantia* samples.
