# Supplementary material for: Whole-genome sequencing provides insights into the genetic diversity and domestication of bitter gourd (Momordica spp.)
Source: Hortic Res. 2020 Jun 1;7:85. doi: 10.1038/s41438-020-0305-5 (PMC7261802; doi:10.1038/s41438-020-0305-5)
Supplement: Supplementary file 2 — Supporting information S2 tables [file 41438_2020_305_MOESM2_ESM.docx]

# Supplementary Tables

## Table S1. Estimation of two bitter gourd genome size based on K-mer statistics.

| **Sample** | **K-mer** | **K-mer number** | **Peak depth** | **Genome size** | **Used base** | **Used read** | **Coverage** |
| --- | --- | --- | --- | --- | --- | --- | --- |
| Dali-11 | 17 | 14,993,541,395 | 50 | 299,870,827 | 18,772,506,844 | 224,946,613 | 61.86 |
| TR | 17 | 32,178,510,001 | 107 | 300,733,738 | 37,415,936,705 | 327,339,169 | 124.41 |

## Table S2. Summary of bitter gourd genome Dali-11 sequencing data.

| **Pair-end Libraries** | **Insert size(bp)** | **Average reads length(bp)** | **Raw** | | | **Clean** | | |
| --- | --- | --- | --- | --- | --- | --- | --- | --- |
|  |  |  | **Total data(Gb)** | **Sequence** | **Physical** | **Total data(Gb)** | **Sequence** | **Physical** |
|  |  |  |  | **depth(×)** | **depth(×)** |  | **depth (×)** | **depth* (×)** |
| Solexa Reads | 170 | 100_100 | 11.9 | 39.67 | 33.84 | 10.91 | 36.37 | 31 |
|  | 170 | 90_90 | 10.29 | 34.3 | 32.51 | 9.21 | 30.7 | 29.09 |
|  | 500 | 100_100 | 13.66 | 45.53 | 114.21 | 12.28 | 40.93 | 102.64 |
|  | 500 | 90_90 | 12.83 | 42.77 | 119.23 | 11.59 | 38.63 | 107.72 |
|  | 800 | 90_90 | 12.19 | 40.63 | 180.28 | 10.83 | 36.1 | 160.91 |
|  | 2000 | 49_49 | 14.79 | 49.3 | 1009.72 | 10.73 | 35.77 | 732.06 |
|  | 5000 | 49_49 | 8.83 | 29.43 | 1506.55 | 5.81 | 19.37 | 991.94 |
|  | 10000 | 49_49 | 4.23 | 14.1 | 1444.81 | 2.62 | 8.73 | 892.53 |
|  | 20000 | 49_49 | 3.79 | 12.63 | 2585.41 | 1.35 | 4.5 | 920.63 |
| Total | - | - | 92.46 | 308.2 | 7026.57 | 75.31 | 251.03 | 3968.52 |
| * Assumed genome size to be 300 Mb. | | | | | | | | |

## Table S3. Summary of bitter gourd genome TR sequencing data.

| **Pair-end libraries** | **Insert size(bp)** | **Average reads length(bp)** | **Raw** | | | **Clean** | | |
| --- | --- | --- | --- | --- | --- | --- | --- | --- |
|  |  |  | **Total data(Gb)** | **Sequence** | **Physical** | **Total data(Gb)** | **Sequence** | **Physical** |
|  |  |  |  | **depth(×)** | **depth(×)** |  | **depth (×)** | **depth* (×)** |
| Solexa Reads | 270 | 125 | 47.41 | 157.51 | 284.45 | 38.53 | 128.01 | 231.17 |
|  | 800 | 125 | 4.97 | 16.55 | 105.94 | 3.95 | 13.18 | 84.34 |
|  | 800 | 125 | 6.57 | 21.9 | 140.17 | 5.38 | 17.93 | 114.74 |
|  | 2000 | 150 | 4.99 | 16.58 | 221.6 | 3.5 | 11.63 | 155.74 |
|  | 5000 | 150 | 3.73 | 12.39 | 414.84 | 2.62 | 8.7 | 291.45 |
|  | 10000 | 150 | 2.88 | 9.57 | 640.04 | 1.69 | 5.61 | 376 |
| Total | - | - | 70.55 | 234.39 | 1807.04 | 55.68 | 184.98 | 1253.44 |
| * Assumed genome size to be 301 Mb. | | | | | | | | |

## Table S4. Statistics of the final Dali-11 genome assembly.

|  | **Scaffold** | | **Contig** | |
| --- | --- | --- | --- | --- |
|  | **Size (bp)** | **Number** | **Size (bp)** | **Number** |
| N50 | 3,296,283 | 24 | 62,575 | 1,345 |
| N60 | 2,140,377 | 35 | 48,753 | 1,864 |
| N70 | 1,746,425 | 50 | 36,130 | 2,549 |
| N80 | 1,140,132 | 71 | 25,596 | 3,488 |
| N90 | 662,740 | 105 | 14,641 | 4,942 |
| Longest | 11,374,639 | --- | 394,598 | --- |
| Total size | 293,642,520 | --- | 286,737,210 | --- |
| Total number (≥2kb) | --- | 297 | --- | 8,600 |

## Table S5. Statistics of the final TR genome assembly.

|  | **Contig** | | **Scaffold** | |
| --- | --- | --- | --- | --- |
|  | **Size (bp)** | **Number** | **Size (bp)** | **Number** |
| N50 | 16,175 | 5,270 | 611,611 | 94 |
| N60 | 13,188 | 7,223 | 415,567 | 155 |
| N70 | 10,269 | 9,678 | 284,521 | 241 |
| N80 | 7,450 | 12,925 | 169,166 | 375 |
| N90 | 4,474 | 17,786 | 84,772 | 623 |
| Longest | 98,483 | ---- | 4,799,038 | ---- |
| Total Size | 285,351,815 | ---- | 296,262,545 | ---- |
| Total Number(≥2kb) | ---- | 23,789 | ---- | 1,643 |

## Table S6. The mapping statistics of Dali-11 and TR assembly (in Excel file).

## Table S7. Statistics of Dali-11 transcriptome sequencing data.

| **Tissue** | **Clean reads** | **Percent mapped to genome (%)** | **Percent mapped to gene (%)** | **Expressed gene** |
| --- | --- | --- | --- | --- |
| Root | 44,287,058 | 81.52% | 32.87% | 20,623 |
| Stem | 44,411,666 | 85.13% | 41.01% | 20,125 |
| Leaf | 44,482,272 | 85.07% | 37.15% | 19,879 |
| Male flower | 44,537,634 | 85.59% | 42.29% | 20,670 |
| Ovary | 44,564,864 | 87.20% | 39.18% | 19,962 |
| Fruit 6 | 46,142,708 | 85.92% | 45.24% | 20,244 |
| Fruit 12, rep1 | 44368364 | 86.89% | 43.03% | 19,598 |
| Fruit 12, rep2 | 45,756,370 | 84.56% | 41.32% | 19,221 |
| Fruit 18, rep1 | 46,192,066 | 82.12% | 44.28% | 19,329 |
| Fruit 18, rep2 | 44,176,758 | 74.05% | 39.58% | 18,975 |
| Fruit 24, rep1 | 44,203,674 | 73.10% | 39.11% | 18,736 |
| Fruit 24, rep2 | 44,077,758 | 79.16% | 38.95% | 19,006 |
| Total | 537,201,192 |  |  |  |

## Table S8. The mapping statistics of unigenes aligned to Dali-11 and TR assembly (in Excel file).

## Table S9. Comparative analysis of gene annotation of genome assembly among 10 genomes using busco-v3 software (database embryophyta_odb10) (in Excel file).

## Table S10. List of scaffolds anchored to the Dal1-11 pseudochromosomes (in Excel file).

## Table S11. Classification of bitter gourd Dali-11 transposable elements (TEs).

|  | **RepBase TEs** | | **TE Proteins** | | ***De novo*** | | **Combined TEs** | |
| --- | --- | --- | --- | --- | --- | --- | --- | --- |
| **Type** | **Length (bp)** | **Rate of genome (%)** | **Length (bp)** | **Rate of genome (%)** | **Length (bp)** | **Rate of genome (%)** | **Length (bp)** | **Rate of genome (%)** |
| DNA | 2,635,285 | 0.9 | 3,240,603 | 1.1 | 6,688,471 | 2.28 | 8,776,173 | 2.99 |
| LINE | 1,192,469 | 0.41 | 4,381,840 | 1.49 | 4,522,848 | 1.54 | 7,380,408 | 2.51 |
| SINE | 18,803 | 0.01 | 0 | 0 | 29,815 | 0.01 | 47,376 | 0.02 |
| LTR | 23,751,124 | 8.09 | 31,973,255 | 10.89 | 88,536,992 | 30.15 | 93,415,537 | 31.81 |
| Other | 1,979 | 0 | 0 | 0 | 0 | 0 | 1,979 | 0 |
| Unknown | 0 | 0 | 0 | 0 | 21,318,469 | 7.26 | 21,318,469 | 7.26 |
| Total | 27,496,997 | 9.36 | 39,586,637 | 13.48 | 116,179,620 | 39.56 | 121,766,725 | 41.47 |

## Table S12. Classification of bitter gourd TR transposable elements.

|  | **RepBase TEs** | | **TE Proteins** | | ***De novo*** | | **Combined TEs** | |
| --- | --- | --- | --- | --- | --- | --- | --- | --- |
| **Type** | **Length (bp)** | **Rate of genome (%)** | **Length (bp)** | **Rate of genome (%)** | **Length (bp)** | **Rate of genome (%)** | **Length (bp)** | **Rate of genome (%)** |
| DNA | 2,574,417 | 0.87 | 1,268,551 | 0.43 | 8,204,095 | 2.77 | 9,482,902 | 3.2 |
| LINE | 1,129,365 | 0.38 | 1,705,220 | 0.58 | 3,811,649 | 1.29 | 5,331,554 | 1.8 |
| SINE | 15,885 | 0.01 | 0 | 0 | 291,526 | 0.1 | 304,542 | 0.1 |
| LTR | 22,444,885 | 7.58 | 24,364,163 | 8.22 | 95,798,335 | 32.34 | 97,904,553 | 33.05 |
| Other | 1,119 | 0 | 0 | 0 | 0 | 0 | 1,119 | 0 |
| Unknown | 0 | 0 | 0 | 0 | 10,709,061 | 3.61 | 10,709,061 | 3.61 |
| Total | 26,056,036 | 8.79 | 27,334,845 | 9.23 | 114,929,577 | 38.79 | 118,184,798 | 39.89 |

| **Species** | **Content** | **DNA** | **LINE** | **SINE** | **LTR** | **Other** | **Unknown** | **Total** |
| --- | --- | --- | --- | --- | --- | --- | --- | --- |
| *M.charantia* Dali-11 | Length (Bp) | 8,776,173 | 7,380,408 | 47,376 | 93,415,537 | 1,979 | 21,318,469 | 121,766,725 |
|  | % in genome | 2.99 | 2.51 | 0.02 | 31.81 | 0 | 7.26 | 41.47 |
| *M.charantia* OHB3-1 | Length (Bp) | 8,754,296 | 5,175,301 | 165,441 | 78,362,054 | 1,611 | 11,745,776 | 99,212,624 |
|  | % in genome | 3.07 | 1.81 | 0.06 | 27.44 | 0 | 4.11 | 34.74 |
| TR | Length (Bp) | 9,482,902 | 5,331,554 | 304,542 | 97,904,553 | 1,119 | 10,709,061 | 118,184,798 |
|  | % in genome | 3.2 | 1.8 | 0.1 | 33.05 | 0 | 3.61 | 39.89 |
| *C.lanatus* | Length (Bp) | 11,542,956 | 3,697,147 | 351,021 | 108,337,647 | 10,135 | 29,042,541 | 141,393,294 |
|  | % in genome | 3.25 | 1.04 | 0.1 | 30.5 | 0 | 8.18 | 39.8 |
| *C.melo* | Length (Bp) | 27,458,011 | 3,178,702 | 41,220 | 101,536,307 | 5,871 | 21,761,675 | 144,006,329 |
|  | % in genome | 6.75 | 0.78 | 0.01 | 24.95 | 0 | 5.35 | 35.39 |
| *C.sativus* | Length (Bp) | 5,842,052 | 2,390,344 | 238,126 | 22,740,709 | 1,220 | 13,519,087 | 41,057,258 |
|  | % in genome | 2.96 | 1.21 | 0.12 | 11.53 | 0 | 6.85 | 20.81 |

## Table S13. Summary of transposable element content in cucurbit genomes.

## Table S14. Detailed transposable element (TE) analysis in Dali-11, TR, OHB3-1 and other three sequenced cucurbit species (*C.sativus*, *C. lanatus*, and *C. melo*) (in Excel file).

## Table S15. Statistics of predicted protein-coding genes for Dali-11 assembly.

| **Gene set** | | **Number** | **Average transcript length (bp)** | **Average CDS length (bp)** | **Average exon per gene** | **Average exon length (bp)** | **Average intron length (bp)** |
| --- | --- | --- | --- | --- | --- | --- | --- |
| *De novo* | *AUGUSTUS* | 22,726 | 3444.95 | 1203.83 | 5.16 | 233.09 | 538.14 |
| Homolog | *A. thaliana* | 24,700 | 2778.63 | 978.42 | 4.05 | 241.31 | 589.32 |
|  | *C.sativus* | 24,601 | 3114.66 | 1128.01 | 4.45 | 253.52 | 575.93 |
|  | *C. melo* | 25,292 | 3174.02 | 1144.57 | 4.57 | 250.7 | 569.2 |
|  | *C.lanatus* | 36,222 | 1881.25 | 760.45 | 3.28 | 231.64 | 490.96 |
| Transcripts | Transcripts_v1 | 15,590 | 6,803 | 1,366 | 6.08 | 224.39 | 542.72 |
|  | Transcripts_v2 | 17,233 | 5127.74 | 1285.98 | 5.50 | 232.88 | 549.29 |
| Final | | 26,427 | 3715.47 | 1061.16 | 4.59 | 230.87 | 542.36 |

## Table S16. Gene set and annotation of Dali-11 assembly (in Excel file).

## Table S17. Statistics of predicted protein-coding genes for bitter gourd TR.

| **Gene set** | | **Gene number** | **Average mRNA length(bp)** | **Average CDS length(bp)** | **Average exon number** | **Average exon length(bp)** | **Average intron length(bp)** |
| --- | --- | --- | --- | --- | --- | --- | --- |
|  |  |  |  |  |  |  |  |
|  |  |  |  |  |  |  |  |
| *De novo* | *AUGUSTUS* | 23,013 | 3285.84 | 1187.28 | 4.93 | 240.7 | 533.63 |
| Homolog | *A_thaliana* | 25,515 | 2426.57 | 917.48 | 3.68 | 249.29 | 563.01 |
|  | *C_lanatus* | 34,947 | 1863.1 | 765.16 | 3.21 | 238.58 | 497.46 |
|  | *C_melo* | 41,743 | 1653.58 | 761.67 | 2.95 | 258.33 | 457.77 |
|  | *C_sativus* | 30,123 | 2243.75 | 872.64 | 3.65 | 239.4 | 518.37 |
| Final | | 28,827 | 2608.98 | 948.91 | 4.01 | 236.4 | 550.8 |

## Table S18. Gene set and annotation of TR assembly (in Excel file).

## Table S19. Functional annotation of predicted genes for Dali-11 assembly.

|  | **Databases** | **Number** | **Percent (%)** |
| --- | --- | --- | --- |
| Total | | 26,427 | 100 |
| Annotated | Nr | 22,403 | 84.77 |
|  | InterPro | 20,270 | 76.7 |
|  | GO | 14,242 | 53.89 |
|  | KEGG | 15,967 | 60.42 |
|  | Swissprot | 17,130 | 64.82 |
|  | TrEMBL | 21,650 | 81.92 |
|  | Total | 22,526 | 85.24 |
| Unannotated | | 3,901 | 14.76 |

## Table S20. Functional annotation of predicted genes for TR assembly.

|  | **Databases** | **Number** | **Percent (%)** |
| --- | --- | --- | --- |
| Total | | 28,827 |  |
| Annotated | Nr | 24,473 | 84.9 |
|  | InterPro | 21,373 | 74.14 |
|  | GO | 15,063 | 52.25 |
|  | KEGG | 16,945 | 58.78 |
|  | Swissprot | 17,942 | 62.24 |
|  | TrEMBL | 23,659 | 82.07 |
|  | Total | 24,632 | 85.45 |
| Unannotated | | 4,195 | 14.55 |

## Table S21. Summary of syntenic blocks between *M. charantia* and TR, *C. lanatus*, *C. melo*, and *C. sativus*.

| ***M. charantia* VS** | **Number of syntenic blocks** | **Genes per block** | **Number of collinear genes in all blocks** | **Mean block length in *M. charantia* (bp)** |
| --- | --- | --- | --- | --- |
| TR | 897 | 26.2007 | 17,402 | 556,100.55 |
| *C. lanatus* | 992 | 22.0484 | 14,938 | 615,196.76 |
| *C. melo* | 807 | 24.4746 | 14,567 | 627,994.06 |
| *C. sativus* | 922 | 22.6204 | 14,804 | 606,314.62 |
| *M. charantia* | 466 | 10.4657 | 4,877 | 550399.7028 |

## Table S22. Summary of gene family clustering.

| **Species** | **Genes number** | **Genes in families** | **Unclustered genes** | **Family number** | **Unique families** | **Average genes per family** |
| --- | --- | --- | --- | --- | --- | --- |
| *M. charantia* | 26,253 | 21,512 | 4,741 | 15,017 | 468 | 1.43 |
| *C. lanatus* | 23,440 | 21,551 | 1,889 | 14,867 | 211 | 1.45 |
| *C. melo* | 26,221 | 22,179 | 4,042 | 15,132 | 484 | 1.47 |
| *C. sativus* | 23,248 | 19,562 | 3,686 | 15,109 | 71 | 1.29 |
| *C. maxima* | 31,988 | 26,997 | 4,991 | 16,654 | 85 | 1.62 |
| *C. moschata* | 32,109 | 27,330 | 4,779 | 16,891 | 51 | 1.62 |
| *C. pepo* | 27,785 | 25,221 | 2,564 | 15,445 | 79 | 1.63 |
| *L. siceraria* | 22,404 | 19,036 | 3,368 | 14,586 | 50 | 1.31 |
| *J. regia* | 35,980 | 31,887 | 4,093 | 13,331 | 1,145 | 2.39 |

## Table S23. Statistics of orthologous gene number of *M. charantia* and other eight plant species (in Excel file).

## Table S24. Orthologous gene sets of *M. charantia* and other eight plant species (in Excel file).

## Table S25. Specific gene families of *M. charantia* among nine plant species (in Excel file).

## Table S26. Specific gene sets of *M. charantia* among nine plant species (in Excel file).

## Table S27. Summary of the resequencing data for the 187 bitter gourd accessions (in Excel file).

## Table S28. Statistics for aligning the resequencing clean reads to Dali-11 reference genome (in Excel file).

## Table S29. The number of SNPs per sample called from Dali-11 genome (in Excel file).

## Table S30. The number of InDels per sample called from Dali-11 genome (in Excel file).

## Table S31. Statistics for aligning the clean reads to TR genome (in Excel file).

## Table S32. The number of SNPs per sample called from TR genome (in Excel file).

## Table S33. The number of InDels per sample called from TR genome (in Excel file).

## Table S34. The neighbor-joining tree of 189 *Momordica* samples constructed based on combined whole genome SNPs called from Dali-11 and TR assembly (in Excel file).

## Table S35. The neighbor-joining tree of 189 *Momordica* samples constructed based on whole genome SNPs called from Dali-11 assembly (in Excel file).

## Table S36. The neighbor-joining tree of 189 *Momordica* samples constructed based on whole genome SNPs called from TR assembly (in Excel file).

## Table S37. Summary of SNPs and InDels within *M. charantia* and TR population.

|  |  | ***M. charantia*** | **TR** |
| --- | --- | --- | --- |
| Number of samples | | 166 | 21 |
| SNPs |  | | |
|  | Number of SNPs | 6,595,112 | 6,098,414 |
|  | UTR3 | 100,489 | 74,083 |
|  | mRNA | 1,160,473 | 865,516 |
|  | UTR5 | 61,986 | 43,814 |
|  | CDS | 256,550 | 154,656 |
|  | NA(intergenic) | 5,461,647 | 5,250,425 |
| InDels |  | | |
|  | Number of Indels | 995,825 | 955,011 |
|  | UTR3 | 22,558 | 20,988 |
|  | mRNA | 186,400 | 174,598 |
|  | UTR5 | 16,029 | 14,434 |
|  | CDS | 7,755 | 5,927 |
|  | Intron splice | 435 | 357 |
|  | NA(intergenic) | 812,711 | 783,786 |
| Some variation sites were situated in more than one gene would be calculated repeatedly. Thus the total number of SNPs or Indels were less than the sum of amount in mRNA and NA. | | | |

## Table S38. The population differentiation index (*FST*) between different bitter gourd groups.

|  | ***M*. *charantia* vs. TR** | **South Asia vs. Southeast Asia** | **South Asia vs. China** | **Southeast Asia vs. China** |
| --- | --- | --- | --- | --- |
| Average of *F_ST_* | 0.85 | 0.19 | 0.37 | 0.15 |

## Table S39. The statistics of the optimal *K* (in Excel file).

## Table S40. The domestication regions identified in CHN30, SA30, and SEA30 (in Excel file).

## Table S41. The candidate domestication genes identified in CHN30, SA30, and SEA30 (in Excel file).

## Table S42. The pathway of domestication genes identified in CHN30 (in Excel file).

## Table S43. The pathway of domestication genes identified in SA30 (in Excel file).

## Table S44. The pathway of domestication genes identified in SEA30 (in Excel file).

## Table S45. Genes involved in early pathway of cucurbitane triterpenoid biosynthesis in bitter gourd (in Excel file).
